# IL-13 is a driver of COVID-19 severity

**DOI:** 10.1172/jci.insight.150107

**Published:** 2021-08-09

**Authors:** Alexandra N. Donlan, Tara E. Sutherland, Chelsea Marie, Saskia Preissner, Benjamin T. Bradley, Rebecca M. Carpenter, Jeffrey M. Sturek, Jennie Z. Ma, G. Brett Moreau, Jeffrey R. Donowitz, Gregory A. Buck, Myrna G. Serrano, Stacey L. Burgess, Mayuresh M. Abhyankar, Cameron Mura, Philip E. Bourne, Robert Preissner, Mary K. Young, Genevieve R. Lyons, Johanna J. Loomba, Sarah J. Ratcliffe, Melinda D. Poulter, Amy J. Mathers, Anthony J. Day, Barbara J. Mann, Judith E. Allen, William A. Petri

**Affiliations:** 1 Division of Infectious Diseases and International Health, Department of Medicine and; 2 Department of Microbiology, Immunology and Cancer Biology, University of Virginia School of Medicine, Charlottesville, Virginia, USA.; 3 Lydia Becker Institute of Immunology and Inflammation, School of Biological Sciences, University of Manchester, Manchester Academic Health Sciences Centre, Manchester, United Kingdom.; 4 Department Oral and Maxillofacial Surgery, Charité – Universitätsmedizin Berlin, Freie Universität Berlin, Humboldt-Universität zu Berlin, and Berlin Institute of Health, Berlin, Germany.; 5 Department of Laboratory Medicine and Pathology, University of Washington, Seattle, Washington, USA.; 6 Division of Pulmonary and Critical Care Medicine, Department of Medicine and; 7 Department of Public Health Sciences, University of Virginia School of Medicine, Charlottesville, Virginia, USA.; 8 Division of Pediatric Infectious Diseases, Children’s Hospital of Richmond and; 9 Department of Microbiology and Immunology, School of Medicine, Virginia Commonwealth University, Richmond, Virginia, USA.; 10 School of Data Science and Department of Biomedical Engineering University of Virginia, Charlottesville, Virginia, USA.; 11 Science-IT and Institute of Physiology, Charité – Universitätsmedizin Berlin, corporate member of Freie Universität Berlin, Humboldt-Universität zu Berlin, and Berlin Institute of Health, Berlin, Germany.; 12 Integrated Translational Health Research Institute (iTHRIV) and; 13 Department of Pathology, University of Virginia School of Medicine, Charlottesville, Virginia, USA.; 14 Wellcome Trust Centre for Cell-Matrix Research, School of Biological Sciences, Faculty of Biology Medicine and Health, University of Manchester, Manchester Academic Health Sciences Centre, Manchester, United Kingdom.

**Keywords:** COVID-19, Immunology, Cytokines, Innate immunity, Th2 response

## Abstract

Immune dysregulation is characteristic of the more severe stages of SARS-CoV-2 infection. Understanding the mechanisms by which the immune system contributes to COVID-19 severity may open new avenues to treatment. Here, we report that elevated IL-13 was associated with the need for mechanical ventilation in 2 independent patient cohorts. In addition, patients who acquired COVID-19 while prescribed Dupilumab, a mAb that blocks IL-13 and IL-4 signaling, had less severe disease. In SARS-CoV-2–infected mice, IL-13 neutralization reduced death and disease severity without affecting viral load, demonstrating an immunopathogenic role for this cytokine. Following anti–IL-13 treatment in infected mice, hyaluronan synthase 1 (*Has1*) was the most downregulated gene, and accumulation of the hyaluronan (HA) polysaccharide was decreased in the lung. In patients with COVID-19, HA was increased in the lungs and plasma. Blockade of the HA receptor, CD44, reduced mortality in infected mice, supporting the importance of HA as a pathogenic mediator. Finally, HA was directly induced in the lungs of mice by administration of IL-13, indicating a new role for IL-13 in lung disease. Understanding the role of IL-13 and HA has important implications for therapy of COVID-19 and, potentially, other pulmonary diseases. IL-13 levels were elevated in patients with severe COVID-19. In a mouse model of the disease, IL-13 neutralization reduced the disease and decreased lung HA deposition. Administration of IL-13–induced HA in the lung. Blockade of the HA receptor CD44 prevented mortality, highlighting a potentially novel mechanism for IL-13–mediated HA synthesis in pulmonary pathology.

## Introduction

SARS-CoV-2, the infectious agent causing the ongoing global COVID-19 pandemic, is a virus that primarily infects the lower respiratory tract of hosts by gaining entry to cells via the receptor angiotensin converting enzyme 2 (ACE2) facilitated by the transmembrane receptor neuropilin-1 (1, 2). The clinical course following infection varies widely from asymptomatic carriage to life-threatening respiratory failure and death.

Since early in the pandemic, it was recognized that patients with severe forms of the disease, i.e., requiring hospitalization or ventilation, exhibited elevated levels of inflammatory cytokines (3). This inflammatory state was associated with end-organ damage and in some cases death (4, 5). While it remains unclear how the individual cytokines associated with this response may be involved in severe outcomes in patients, inflammation is thought to be a primary driver of later stages of this disease. In support of this hypothesis, the use of the anti-inflammatory steroid dexamethasone decreased mortality by 29% in patients with COVID-19 who required mechanical ventilation (6).

Aligned with these clinical observations, efforts to characterize the host response to infection and identify contributors to severe clinical outcomes have been ongoing since the pandemic began. Proinflammatory mediators such as the cytokines interleukin-6 (IL-6) and TNFα have been associated with severe disease. Cytokine-targeted therapies have been proposed and in some cases are in clinical trials. For example, the recently completed Adaptive COVID-19 Treatment Trial 2 (ACTT-2) showed a faster time to recovery with remdesivir plus the Janus kinase inhibitor baracitinib compared with remdesivir alone (7), as well as ACTT-4, which compares baracitinib plus remdesivir with dexamethasone plus remdesivir (8).

Descriptive studies of the immune response to SARS-CoV-2 have shown it to be highly heterogeneous (9–11), including the observations that CD4^+^ T cells from patients with COVID-19 secreted the Th1 cytokine IFN-γ, the Th17 cytokines IL-17A and IL-17F, and the Th2 cytokine IL-4 (12, 13). This level of diversity and variability make it especially challenging to find specific drivers of the disease and options for therapies. Consequently, understanding the mechanisms by which distinct immune responses contribute to COVID-19 severity will be crucial to designing personalized or targeted interventions, and, ultimately, to improving upon the current steroid-based treatments.

In this study, we characterized the immune response of patients with COVID-19 and identified the type 2 cytokine, IL-13, as associated with severe outcomes. Using a mouse model of COVID-19, we discovered that IL-13 promotes severe disease, and that this response is likely to be at least partially mediated by the deposition of HA in the lungs.

## Results

### IL-13 is associated with severe COVID-19 in 2 patient cohorts.

**Figure 1:**
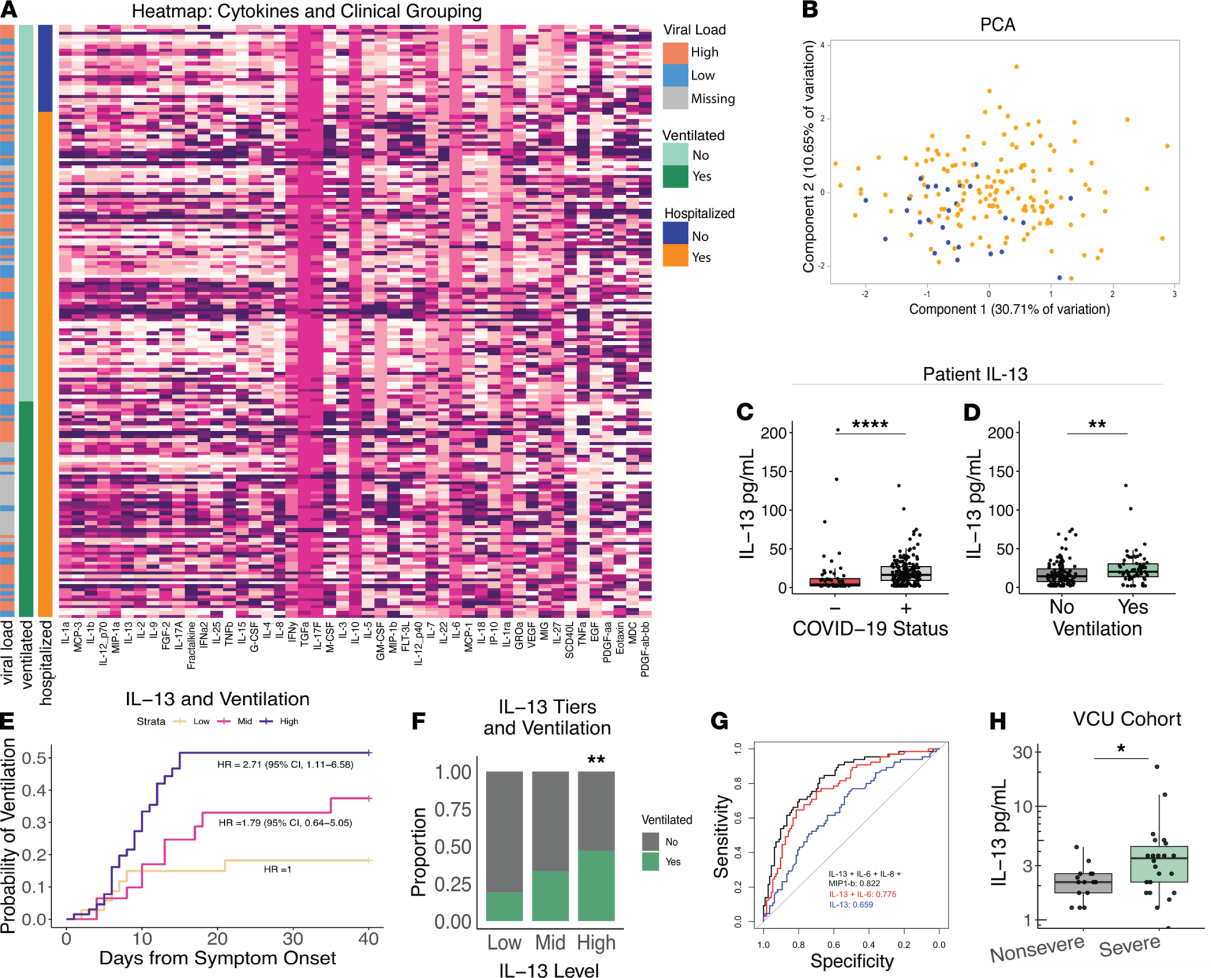
Type 2 immune response in patients with severe COVID-19 disease. (**A**–**E**) Cytokines were measured in plasma from 26 outpatients and 152 inpatients with COVID-19 infection at the University of Virginia Hospital using a 48-plex cytokine array. (**A**) Heatmap of plasma cytokines, supplemental oxygen requirement and nasopharyngeal viral load, with rows ordered by patient status (outpatient (OP) versus inpatient (IP)) and columns by cytokine PC 1 which included IL-13 (Supplemental Table 2). (**B**) Scatterplot comparing PC 1 and 2 from PCA of the plasma cytokines (orange inpatients and blue outpatients). (**C**) Plasma IL-13 levels in patients with COVID-19 who were or were not diagnosed with COVID-19 or (**D**) did or did not require mechanical ventilation (Wilcox test). Data shown by box-and-whisker plots representing the median, interquartile range (box), upper and lower quartiles (whiskers), and outliers as points falling outside the bounds of the upper and lower quartiles. (**E**) Kaplan-Meier survival analysis of the relationship between IL-13 level and mechanical ventilation. Comparison made to lowest IL-13 quantile (Cox proportional hazards test adjusted for age, sex, and comorbidities). (**F**) Proportion of patients with COVID-19 requiring mechanical ventilation stratified by IL-13 plasma cytokine levels (χ^2^ analysis). (**G**) ROC curve with AUC plotted from: IL-13 alone (blue); IL-13 and IL-6 (red); or IL-13, IL-6, IL-8, and MIP-1b (black). (**H**) IL-13 levels in 19 nonsevere and 26 severe (requiring supplemental oxygen) patients with COVID-19 from Virginia Commonwealth University Hospital (Wilcox test). *P < 0.05; **P < 0.005; ****P < 0.0001.

We analyzed plasma cytokines in 178 patients with COVID-19 at the University of Virginia Hospital, 26 of whom received their care as outpatients and 152 as inpatients (Supplemental Table 1; supplemental material available online with this article; https://doi.org/10.1172/jci.insight.150107DS1). Cytokines were measured in the plasma sample taken closest to the first positive COVID-19 quantitative PCR (qPCR) test (Supplemental Figure 1). To understand the potential interrelationships between the different cytokines measured in our cohort, we generated a heatmap for patients, grouped by hospitalization and ventilation status (Figure 1A). While ventilated patients appeared to have elevated levels (dark purple) of many of the measured cytokines, there was a high degree of heterogeneity among the patients. Cytokines were arranged by principal component 1 (PC1) from Figure 1B.

The scatterplot of PC1 and PC2 from a principal coordinate analysis (PCA; Figure 1B) showed a separation, albeit with overlap, of outpatients with less severe disease, (blue dots) from inpatients (yellow dots). IL-13 was in the top 6 of the 47 cytokines/growth factors in PC1 of the PCA (Supplemental Table 2). This is important because the components estimated via PCA are able to retain information, separating patients by disease severity (inpatient versus outpatient). IL-13’s position as a high-ranking contributor to PC1 suggested its overall importance in the disease process. Additionally, network analysis of all of the cytokines measured showed the close relationship between IL-13 and other type 2 cytokines identified in PC1, including IL-9 and IL-25 (Supplemental Figure 2A).

IL-13 is implicated in numerous processes, including a) recruitment of eosinophils and M2 macrophages to the lung, b) induction of mucus secretion into the airways and goblet cell metaplasia, c) proliferation of smooth muscle cells, and d) fibrosis via fibroblast activation and subsequent collagen deposition (14, 15). Therefore, IL-13, as an integral orchestrator of pathogenic responses in the lung, was of particular interest to us. Plasma levels of IL-13 were significantly higher in patients who were positive with COVID-19 compared with uninfected patients (Figure 1C), consistent with previous reports (16–18). Moreover, we found plasma IL-13 levels were also significantly elevated in patients who required mechanical ventilation (Figure 1D). Additionally, when stratified into 3 IL-13–expression level groups, patients in the higher-expressing tiers were more likely to be ventilated and the risk of ventilation was 2.7 times greater for those in the top tier compared with those in the lowest tier (HR 2.71; 95% CI, 1.11–6.58; Figure 1, E and F). To determine whether this association with IL-13 and disease severity was because patients who required ventilation had elevated cytokines due to a longer duration of illness by the time their sample was taken, we performed a linear regression between IL-13 and the days from symptom onset to blood draw (Supplemental Figure 2B) and saw no significant correlation. However, we did observe that IL-13 plasma levels increased from patients’ initial to secondary blood draw, on average (Supplemental Figure 3), suggesting that levels of IL-13 could be increasing within an individual patient’s disease course. To assess the ability of IL-13, alone or in combination with other cytokines, to be able to predict ventilation outcomes in patients, we performed a receiver operating characteristic (ROC) analysis. We found that IL-13 alone performed modestly (AUC = 0.659). Inclusion of the cytokine IL-6 increased the predictive capability (AUC = 0.775) and the additional inclusion of IL-8 and Macrophage inflammatory protein–β (MIP-β) further improved the model (AUC = 0.822; Figure 1G). To validate results from the University of Virginia Hospital, cytokines from an additional 48 inpatients with COVID-19 from Virginia Commonwealth University Medical Center were analyzed (Supplemental Figure 1B and Supplemental Table 3). IL-13 levels measured in plasma were found to be elevated in the hospitalized patients who received oxygen via high flow nasal canula (HFNC) or mechanical ventilation compared with inpatients who did not (Figure 1H).

Consistent with our study, Lucas et al. (19) found that IL-13 increased from day 5–20 of illness in patients with severe COVID-19 requiring ICU and/or mechanical ventilation. Together, these data highlight that IL-13 may be an important component of host responses to SARS-CoV-2 infection and could be driving severe disease.

### Type 2 immunity is induced in k18-hACE2 mice.

To test the contribution of IL-13 to respiratory failure in COVID-19, we utilized a K18-hACE2 transgenic mouse model of COVID-19 (20–22). In this model, mice progress to severe disease starting at day 5 after infection with SARS-CoV-2, with most mice succumbing to infection by day 7 or 8.

**Figure 2:**
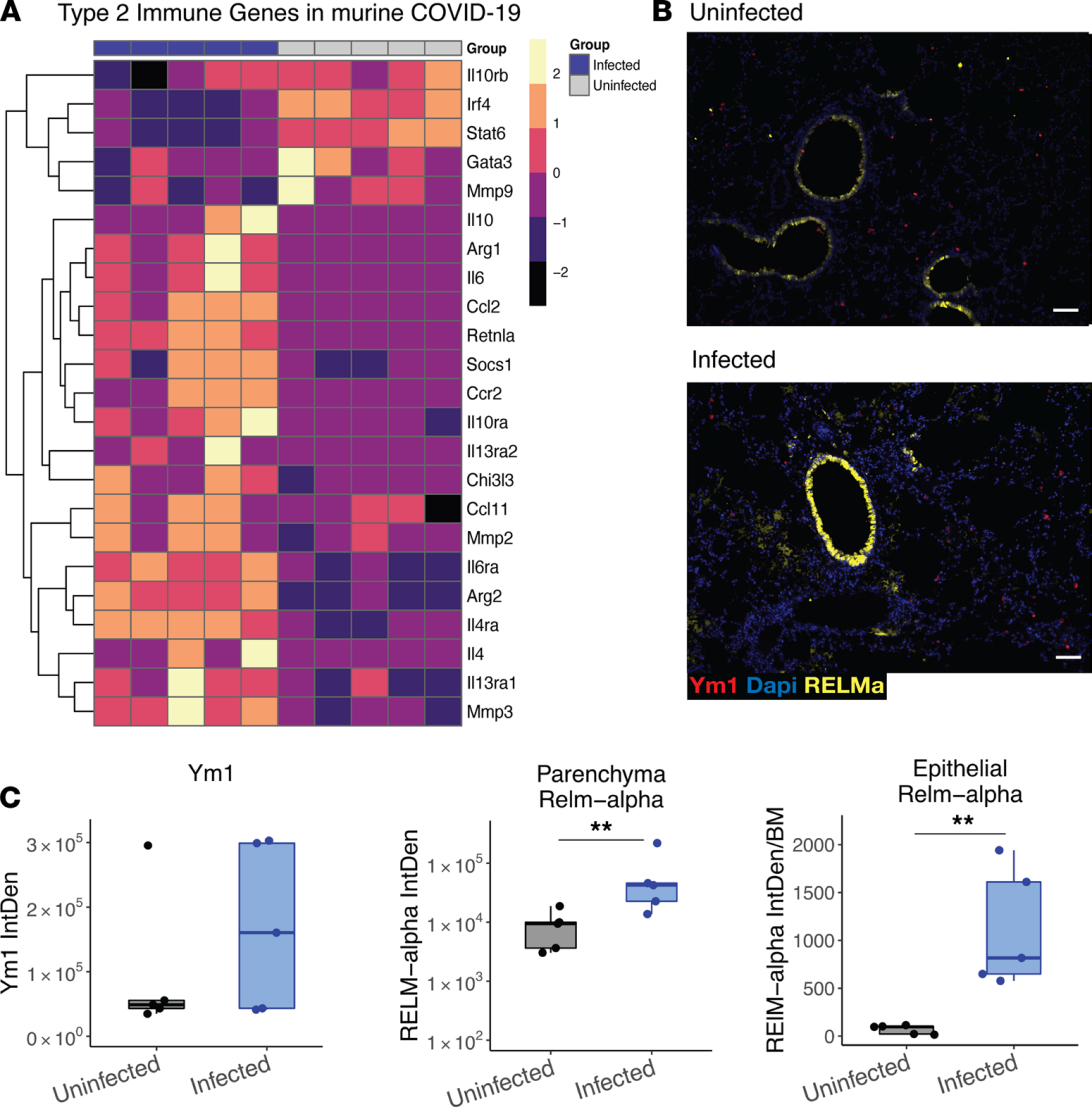
Type 2 immune response in lungs of mice following infection with SARS-CoV-2. Ten-week-old male mice (Tg K18-hACE2 2Prlmn) were infected with 5 × 10^3^ PFU of SARS-CoV-2 and lung tissue examined on day 5 after infection by RNA-Seq and IHC. (**A**) Type 2 gene expression in the lungs of infected versus uninfected mice (heatmap of normalized values of manually curated list of type 2 immune pathway genes). (**B**) IHC of the type 2 immunity proteins RELMα and Ym1 in the lungs of infected and uninfected mice. (**C**) Quantification of IHC scoring for RELMα and Ym1 (mixed effect model). Scale bar: 70 μm. *n =* 5 mice/group. **P < 0.005.

We characterized the impact of SARS-CoV-2 infection in the mouse lung. Reactome analysis of differentially regulated genes from whole tissue RNA-Seq revealed a significant enrichment of genes involved in IL-4 and IL-13 signaling in the infected lung (FDR adjusted *P* value = 0.03) on day 5 after infection (Supplemental Table 4). Importantly, *Il4ra* and *Il13ra1* were upregulated, indicating the potential for increased signaling even in the absence of detectable increases in cytokine expression. Additional type 2 immune effectors known to be regulated by IL-4 and IL-13, including *Chil3*, *Retnla*, *Ccl11* and *Arg1*, were impacted by SARS-CoV-2 infection (Figure 2A).

In support of enhanced type 2-associated genes, protein expression of Ym1 (*Chil3*) and Resistin-like Molecule α (RELMα; *Retnla*) was increased in the lungs following infection (Figure 2, B and C), as measured by IHC. Together, this highlighted that many components of type 2 immunity were induced in the lungs due to infection. It is important to note that IL-4 and IL-13 share a receptor subunit and induce common pathways, so it is difficult to delineate their respective contributions to the upregulation of type 2 effector genes (23). However, because we observed an association of IL-13 but not IL-4 (Supplemental Figure 1) with severe disease in patients, we hypothesized that IL-13 signaling in the lung following infection was contributing to worse outcomes.

### IL-13 neutralization in mice reduces disease severity.

**Figure 3:**
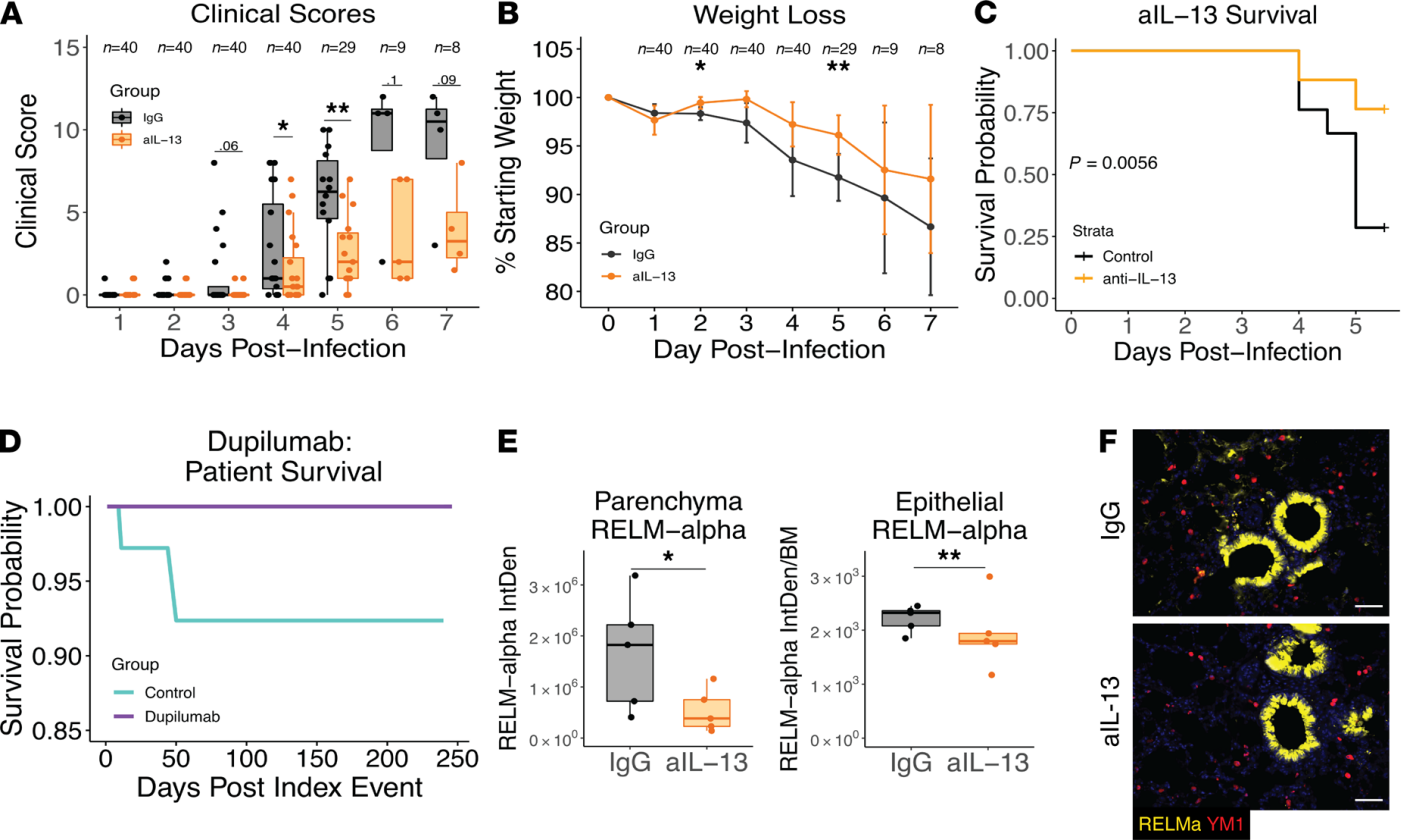
IL-13 neutralization protects from severe COVID-19 in K18-hACE2 mice and dupilumab use is associated with protection in humans. Mice were infected on day 0 with 5 × 10^3^ PFU of SARS-CoV-2 and administered 150 μg of anti–IL-13 or an IgG isotype control antibody i.p. on days 0, 2, and 4. (**A**) Clinical scores of illness severity on days 1–7 after infection. Clinical scoring was measured by weight loss (scores 0–5), posture and appearance of fur (piloerection) (scores 0–2), activity (scores 0–3), and eye closure (scores 0–2). (**B**) Weight loss on days 1–7 after infection. (**A** and **B** Student’s *t* test). (**C**) Kaplan-Meier survival analysis in mice. (**D**) Kaplan-Meier curve generated from data obtained from TriNetX: 1:1 matching based on 81 patients who had been prescribed Dupilumab independently of their COVID-19 diagnosis. (**E**) Quantification of intensity of staining for parenchyma and epithelial RELMα following IL-13 neutralization (log-transformed, mixed effect model). (**F**) IHC of lung tissue stained for RELMα (yellow) in parenchyma or airway, and DAPI (blue). (*n =* 5 mice/group; (**A**–**C**) combined from 3 independently conducted experiments).

To directly test whether IL-13 was deleterious following SARS-CoV-2 infection, we administered i.p. injections of anti–IL-13 or isotype-matched control IgG on days 0, 2, and 4 after infection. Infected mice receiving anti–IL-13 had significantly reduced symptoms as measured by clinical scores (ref. 19 and Figure 3A), weight loss (Figure 3B), and mortality (Figure 3C), demonstrating a pathogenic role for this cytokine during disease. Importantly, anti–IL-13 did not alter viral load in the lungs on day 5 (Supplemental Figure 4A), suggesting disease amelioration was not due to a reduced infectious burden but likely due to events downstream of IL-13 signaling.

Dupilumab is a monoclonal antibody that blocks IL-13 and IL-4 signaling. It is directed against the IL-4Rα subunit that is shared with the IL-13 receptor (24). Based on our findings, we considered the possibility that patients prescribed Dupilumab for asthma, atopic dermatitis or allergic sinusitis may be protected from severe COVID-19. To test this hypothesis, we conducted a retrospective analysis of a large international COVID-19 cohort comprised of 350,004 cases, 81 of whom had been prescribed Dupilumab prior to and independently of their COVID-19 diagnosis (Supplemental Table 5). We generated a subcohort using 1:1 propensity score matching as well as an additional subcohort for patients with diagnosed asthma, atopic dermatitis, or rhinosinusitis, for which Dupilumab is prescribed. Importantly, Dupilumab use was associated with a lower risk of ventilation and death from COVID-19 (Figure 3D and Table 1). To assess whether Dupilumab was associated with clinical proxies of inflammation, we also examined levels of C-reactive protein (CRP), an acute phase protein that increases during inflammation and correlates with poor outcomes in COVID-19 (25). CRP levels were reduced in Dupilumab-prescribed patients with COVID-19 (Table 2), suggesting that blocking type 2 immunity may lower overall disease pathology and increase survival rates. We also examined Dupilumab and COVID-19 in the National COVID Cohort Collaborative (N3C) as a validation cohort. The 31 patients who contracted COVID-19 while on Dupilumab also had a lower rate of hospitalization, ventilation, and death compared with matched controls (Supplemental Table 6).

To further understand the potential mechanism by which IL-13 exacerbated disease, we used the mouse model to ask whether the reduction in disease severity following IL-13 neutralization corresponded with changes in the lung tissue. We therefore assessed histological parameters of pathology by H&E staining. We have previously reported that infection with SARS-CoV-2 in this model results in lung damage (20); however, IL-13 blockade resulted in little change in lung injury (Supplemental Figure 4, B and C). In contrast, goblet cell metaplasia was subtly, albeit significantly, induced following infection, and this increase was reduced by neutralization of IL-13 (Supplemental Figure 4D). During type 2 inflammation in the lung, IL-13 is a significant driver of goblet cell responses (14), and our data provide evidence that IL-13 signaling is active in COVID-19 but less dramatic, histologically, than in other models of type 2 immunity (26, 27).

To further investigate how IL-13 neutralization protected from COVID-19, we assessed expression of the type 2 associated proteins Ym1 and RELMα. Fluorescence microscopy of the lung revealed a significant decrease in RELMα following neutralization of IL-13, which was evident in both the parenchyma and within the epithelial cells (Figure 3, E and F). However, no change in Ym1 following IL-13 neutralization was detected (Supplemental Figure 4E). In addition, no overt changes in cytokine levels or cell composition in the bronchoalveolar lavage fluid (BALF) were observed (Supplemental Figure 4, F and G). We concluded that the mechanism by which IL-13 was promoting more severe COVID-19 was not necessarily through the type 2 pathways typically observed in the lung.

### Hyaluronan is associated with severe COVID-19.

**Figure 4:**
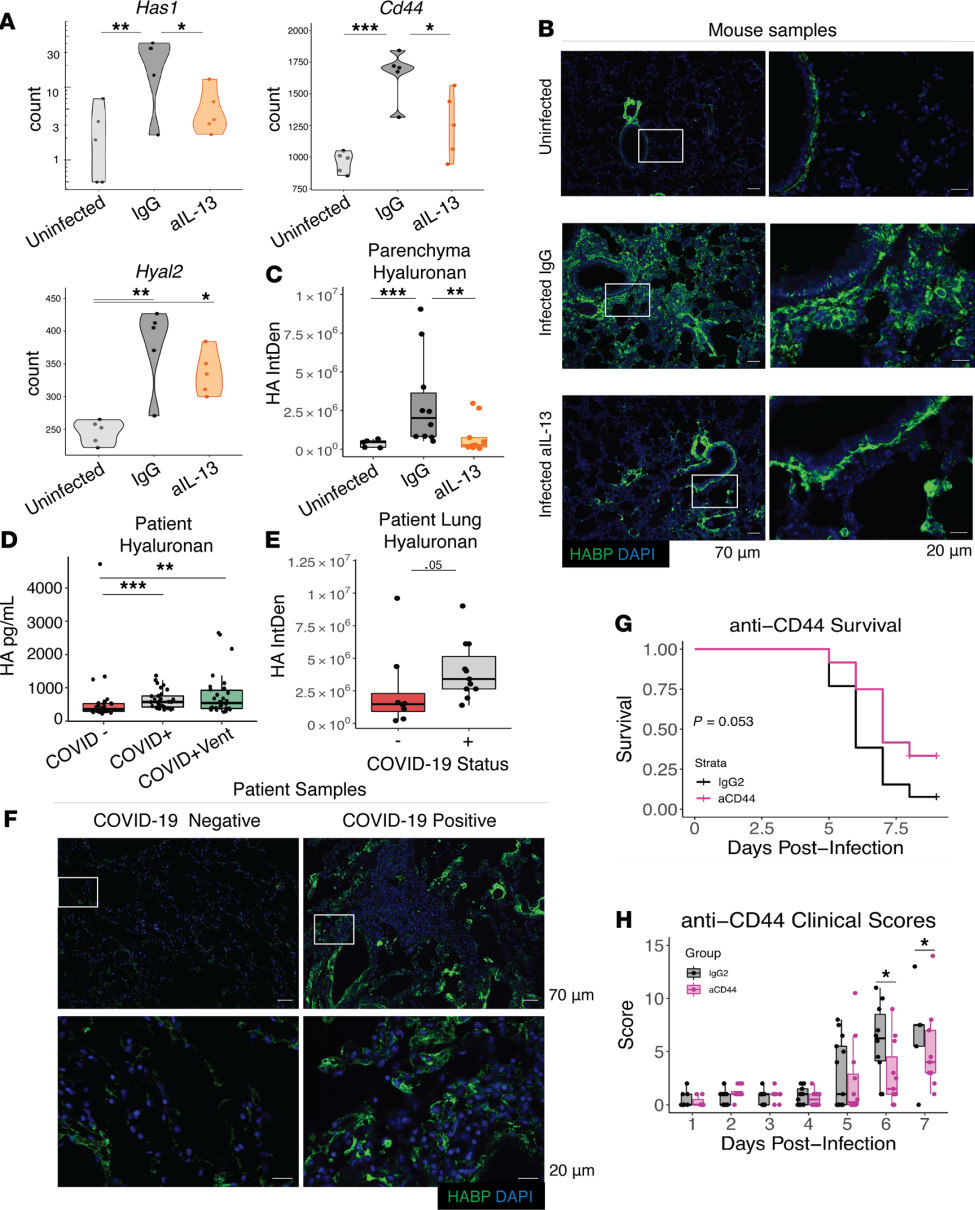
HA and COVID-19 disease. Mice received i.p. injections of anti–IL-13 on days 0 and 2 after infection, were euthanized on day 5, and lung tissue was split and placed either into trizol tissue reagent for RNA analysis or formaldehyde for paraffin embedding and IHC. (**A**) Gene expression in mouse lung of *Has1*, the HA receptor *Cd44,* and Hyaluronidase 2 (*Has2*) of infected mice with anti–IL-13, isotype control antibody and uninfected controls (Tukey’s HSD). (**B**) Staining of HA in mouse lung (with HABP); Scale bar: 70μm. Rectangle indicates area magnified in image to left; Scale bar: 20μm. (**C**) Quantification of HA deposition in tissue following infection and neutralization of IL-13 (mixed effect model; combined 2 experiments). (**D**) Hyaluronan was measured in the plasma of COVID-19–negative controls and in patients with COVID-19 that did or did not require supplemental oxygen. Postmortem lung samples were obtained from fatal COVID-19 cases and control tissue from COVID-19 negative deaths. (**E**) Quantification of HA deposition in fatal COVID-19 disease (*n =* 11) and controls (*n =* 8) (log-transformed, mixed effect model) using HABP. (**F**) Representative images of staining for HA (with HABP) from human lung samples; Scale bar: 70μm. Rectangle indicates area magnified in image below; Scale bar: 20μm. Mice were administered anti-CD44 or IgG2 isotype control on each of day 1–4 after infection. (**G**) Kaplan-Meier survival curve (log-rank) and (**H**) Clinical scores for mice (Student’s *t* test); combined 2 independent experiments. Hyaluronan-binding protein, HABP. *n =* 5 mice/group. *P < 0.05; **P < 0.005; ***P < 0.0005.

Given the above, we took an unbiased approach to evaluate the impact of IL-13 during COVID-19. RNA-Seq analysis was performed on whole lung tissue from IL-13–neutralized and control-treated mice at day 5 after infection to evaluate transcriptional responses downstream of IL-13. Intriguingly, this analysis identified the enzyme Has1, which was upregulated during infection, as the most downregulated gene following IL-13 neutralization (Table 3 and Figure 4A). In addition to Has1, other genes associated with the signaling or synthesis of the HA polysaccharide, Cd44, (Figure 4A) and Has2 (Supplemental Figure 5A), respectively, were also downregulated following IL-13 neutralization. Additionally, hyaluronidases, which break down HA that has been endocytosed, were upregulated during infection (Figure 4A and Supplemental Figure 5A), altogether supporting a potential role for HA during COVID-19. Deposition of this polysaccharide was found to be significantly increased in SARS-CoV-2–infected mice compared with uninfected mice, specifically in the parenchyma of the lungs (Figure 4, B and C). Following IL-13 neutralization in infected mice, HA deposition was significantly reduced in the parenchyma (Figure 4, B and C, and Supplemental Figure 5B).

Our finding in the mouse model that IL-13 regulates the HA pathway in COVID-19 was noteworthy as there is evidence to support a pathological role for HA in humans with lung disease (28), including COVID-19 (29–33). We observed that patients infected with SARS-CoV-2 had higher plasma levels of HA (Figure 4D). Additionally, HA was elevated in the postmortem lung tissue from patients who died of severe COVID-19 (Figure 4, E and F), supporting prior observations (30). It is important to note, however, that HA can be elevated due to an array of factors, including occupation, respiratory diseases, or other infections unrelated to SARS-CoV-2 (34), which likely accounts for the variation in patients who had tested negative for COVID-19. One uninfected patient, in particular, with high levels of HA, had died due to cardiac arrest while experiencing a postoperative wound infection, both of which could have resulted in the increased HA that we observed.

In a murine influenza model, administering hyaluronidase to break down pathogenic HA resulted in ameliorated disease (28). We followed a similar approach to test the impact of HA during COVID-19, by administering hyaluronidase i.n. on day 5 after infection. This resulted in modest, albeit not statistically significant, protection from weight loss and clinical scores (Supplemental Figure 5, C–E). This could suggest that IL-13–dependent accumulation of HA may not act alone to drive severe disease, or that timing of hyaluronidase administration is important for the effect this treatment may have. Importantly, however, ovine hyaluronidase used here also reduces chondroitin sulfate (35), which may have unpredictable effects. We therefore pursued this observation by testing if blockade of the HA receptor CD44, which was also downregulated by IL-13 neutralization (Figure 4A), would be protective. This receptor is present on multiple cell types, including inflammatory cells, that may utilize CD44-HA interactions for migration, activation, proliferation, or other functions that could contribute to pathogenic responses (36, 37). Blockade of CD44 from days 1–4 after infection resulted in improved clinical survival and clinical scores (Figure 4, G and H, and Supplemental Figure 5F).

**Figure 5:**
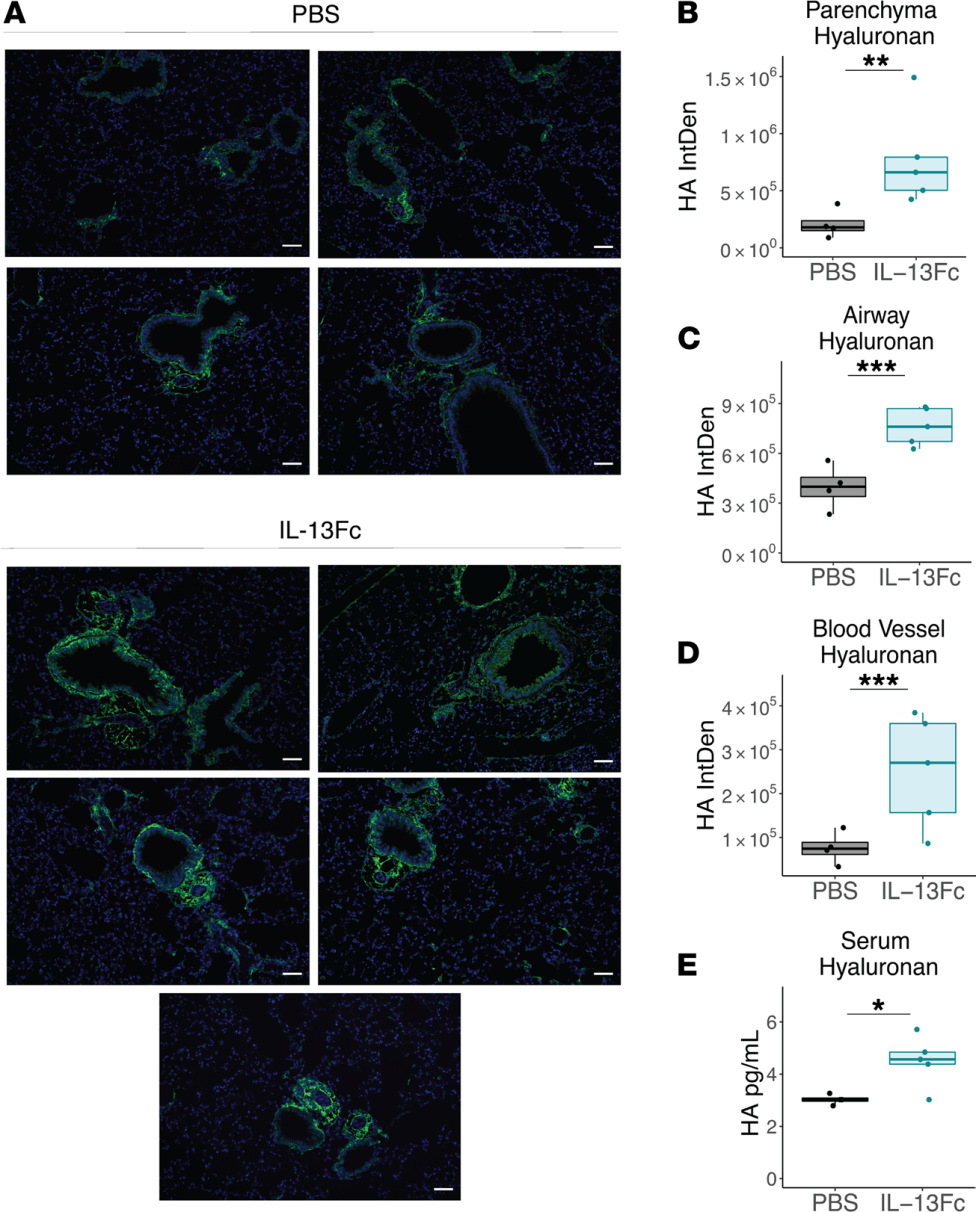
IL-13 administration promotes HA accumulation in mice. Uninfected mice were administered IL-13 Fc (*n =* 5) or PBS (*n =* 4) on days 0 and 2 and lung tissue and serum was collected 24 hours later. Lung tissue was sectioned and stained for HABP. (**A**) Representative images from each mouse. Quantification of HAPB staining in the (**B**) parenchyma, (**C**) airway, and (**D**) blood vessels for quantification of HA deposition in the tissue. (**B**–**D**; mixed effect model). (**E**) Hyaluronan was measured in the serum by ELISA (Student’s *t* test). Hyaluronan-binding protein, HABP. Scale bar: 70μm. *P < 0.05; **P < 0.01; ***P < 0.001.

Because the finding that neutralization of IL-13 during COVID-19 led to reduced HA deposition was potentially novel, we investigated whether IL-13 administration could directly result in increased HA deposition in the lungs of mice. To uninfected mice, we administered IL-13 Fc i.n. on days 0 and 2 before collecting lung tissue and sera on day 3. This resulted in significantly increased deposition of HA in the tissue by IHC (Figure 5, A–D) and increased levels of HA in the serum (Figure 5E), supporting the discovery that IL-13 can regulate HA deposition and accumulation in the lungs. Together, these data indicated that HA production and signaling are downstream of IL-13 and contribute to disease outcome in mice and patients.

## Discussion

Here, we have shown that the type 2 cytokine, IL-13, is associated with severe COVID-19. The IL-13 blocking drug, Dupilumab, in turn, is associated with better outcomes in patients with COVID-19. Additionally, neutralization of IL-13 in mice infected with SARS-CoV-2 protects from death, in part by blocking excessive HA synthesis and excessive deposition. Overall, this work opens a new avenue in the study of COVID-19 by demonstrating a causal role for type 2 immune responses and downstream HA accumulation in respiratory failure and offers potential paths for immunotherapy of this disease. Considering the extreme heterogeneity in immune responses to COVID-19, it is unlikely that IL-13 blockade will work in all patients. Nonetheless, understanding the underlying mechanism and identifying those most likely to benefit from such a treatment would be a major advance.

The mechanism through which IL-13 promoted severe disease was challenging to identify, as there was only a modest impact on the downstream effectors of IL-13 that are commonly seen during allergic or asthmatic inflammation. Although there were decreases in type 2-associated responses, e.g., goblet cells and RELMα following IL-13 blockade, the biological significance of their contribution was likely minimal given the low magnitude of their induction compared with other models of type 2 immunity in the lung. Additionally, periostin, a marker for asthma responses in the lung (38), was also decreased following IL-13 neutralization (data not shown), suggesting the neutralization of IL-13 was affecting typical downstream pathways. However, it was not increased following infection, indicating that asthmatic-like responses such as eosinophilia or tissue remodeling were not occurring by day 5 after infection.

Notably, in our RNA-Seq data set, Stat6 and Gata3 were not upregulated during COVID-19 in mice, which may reflect an inability to detect changes in these mRNAs at that time point. However, it is also possible that some type 2-associated genes are driven by other type 2 mediators such as STAT3, which is upregulated following infection (Figure 2A and refs. 39, 40). Since STAT3 is involved downstream of other cytokines, such as IL-6 and IL-10, this could implicate a complex interplay of these responses and would be consistent with our finding that responses downstream of IL-13 did not follow canonical type 2-mediated pathology.

The identification of *Has1* as the most downregulated gene following IL-13 neutralization in infected mouse lungs, along with downregulation of *Has2* and *Cd44,* 2 other genes involved in the HA pathway, enabled the discovery of a potentially novel route by which IL-13 impacts pathology via upregulation of HA synthesis during infection. We showed that IL-13 neutralization not only decreased *Has1* gene expression and lowered HA levels/deposition, but IL-13 administration also directly increased HA accumulation in the lung.

Downstream of HA production, neutralization of the HA receptor, CD44, improved survival in infected mice. Previous work has shown that IL-13 can result in the induction of CD44 (41, 42), and elevated CD44 has been found in patients with asthma (43). While the relationship between CD44 and IL-13 is still not fully characterized, these observations highlight the ability for CD44 to be regulated by IL-13 and other components of type 2 inflammation. While our work stops short of a full mechanistic understanding of the function of HA in COVID-19, it is interesting to speculate that this polysaccharide may contribute to inflammation in the lung by providing a matrix for inflammatory cells to migrate over and adhere to, as well as via signaling through, its receptor CD44. Additionally, excessive build-up of HA, which binds a large amount of water, could contribute to severely impaired oxygen uptake or result in edema, which are significant components of disease in hospitalized patients.

Because increases in HA have been observed in patients with COVID-19, this study provides a potential link between the association of IL-13 with severe disease (19) and increased HA seen in other studies (29, 30). Understanding the relationship between IL-13 and HA may be widely relevant to respiratory diseases beyond COVID-19.

Neutralization of IL-13 also resulted in changes to other genes that may be of interest in future studies on the contributions of IL-13 to lung inflammation during COVID-19. *Arg1*, the gene that encodes for Arginase-1 (Arg1), was significantly downregulated following anti–IL-13 treatment. Arg1 expression is often utilized as a marker for alternatively activated macrophages (AAMs), which IL-13 is known to promote. Additionally, RELMα can be a marker of AAMs, and in combination with the observation of decreased Arg1 may suggest that these macrophages could be downstream of IL-13 signaling during COVID-19. The presence of AAMs, or similarly characterized macrophages, has been observed in patients with severe disease (44), together suggesting that IL-13 may promote these cells as a pathogenic mediator for disease and potentially longer-term pathology associated with long COVID-19. While the role for these cells during COVID-19 has not been fully explored, they may contribute to dysregulated immunity, IL-10 production, or other deleterious tissue responses.

Overall, this work in humans and mice implicates IL-13 as an important driver of severe outcomes during COVID-19, in part through HA-CD44 engagement in the lungs. Understanding the pathogenic role of IL-13 in the murine model, combined with the results correlating Dupilumab use with better patient outcomes, emphasizes the potential impact this work may have on improving patients’ lives.

## Methods

### Patients.

Patients with a positive qPCR test for COVID-19 at the Clinical Microbiology Laboratory at the University of Virginia Medical Center had any remnant EDTA-plasma samples collected within 48 hours of the time of diagnosis or hospitalization. EDTA plasma from 178 patients diagnosed from March to September 2020 were analyzed in this study. Blood was centrifuged at x1300 *g* for 10 minutes, then plasma was aliquoted and stored at –80°C until testing.

Demographics (age, sex, race), comorbidities, hospitalization status, lab results, and other clinical information were obtained from the electronic medical record (EMR; Supplemental Table 1). The severity of COVID-19 illness was assessed through review of the EMR in 2 ways: first, by inpatient admission versus outpatient care, and second, by the use of supplemental oxygen (none versus any supplemental oxygen, and supplemental oxygen delineated as low flow nasal canula versus mechanical ventilation or high flow oxygen). In addition, supplemental oxygen was scored as occurring at the time of the blood draw or at a future time. Days from symptom onset were scored as per Lucas et al. (19) based on the patient’s determination or by the earliest reported symptom from the patient as recorded in the EMR.

Cytokine concentrations in plasma were measured using the MILLIPLEX MAP Human Cytokine/Chemokine/Growth Factor Panel A (48 Plex) (MilliporeSigma) by the Flow Cytometry Facility of the University of Virginia. Cytokines detected were sCD40L, EGF, Eotaxin, FGF-2, Flt-3 ligand, Fractalkine, G-CSF, GM-CSF, GROα, IFNα2, IFNγ, IL-1α, IL-1β, IL-1ra, IL-2, IL-3, IL-4, IL-5, IL-6, IL-7, IL-8, IL-9, IL-10, IL-12 (p40), IL-12 (p70), IL-13, IL-15, IL-17A, IL-17E/IL-25, IL-17F, IL-18, IL-22, IL-27, IP-10, MCP-1, MCP-3, M-CSF, MDC (CCL22), MIG, MIP-1α, MIP-1β, PDGF-AA, PDGF-AB/BB, TGFα, TNFα, TNFβ, and VEGF-A (Supplemental Figure 1) (RANTES was excluded). HA was measured using the Hyaluronan Duoset ELISA (R&D Systems, DY3614-05) with plasma diluted 1:50.

As a test of validation of the results from the University of Virginia Hospital, an additional 47 patients with symptomatic COVID-19 (all inpatients) from Virginia Commonwealth University Medical Center were analyzed. All procedures performed in this study were approved by the Virginia Commonwealth University Institutional Review Board and in accordance with the 1964 Helsinki Declaration and its later amendments or comparable ethical standards. Informed consent was obtained from all participants or by their legally authorized representatives if they were unable to give consent. Severe was defined as any patient requiring HFNC oxygen delivery or intubation, or whose disease resulted in sepsis or death. IL-13 was measured in EDTA plasma from these patients using the Bio-Plex Pro Human Cytokine 27-plex Assay (R&D Systems; Supplemental Table 4).

### Database and inclusion criteria for Dupilumab.

Data were retrieved from the COVID-19 Research Network provided by TriNetX, comprising 400 million patients from 130 health care organizations in 30 countries (database access on 12/05/2020). Patients with COVID-19 were identified via the ICD-10 code U07.1 or the presence of a SARS-CoV-2–related RNA diagnosis within the last 11 months. Propensity scores matched cohorts 1:1 using a nearest neighbor greedy matching algorithm with a caliper of 0.25 times the standard deviation. Outcomes were defined as ventilation assist and death. Measures of association including risk differences with their respective 95% CIs were calculated. In addition, Kaplan-Meier curves were generated for each analysis.

A subcohort with indications for Dupilumab use was generated using ICD-10 codes for asthma (J45), atopic dermatitis (L20.8), and pansinusitis (J01.40 and J32.4). Drug use was identified via RxNorm codes for Dupilumab (1876376) and the lab value for C-reactive protein (9063). Patients receiving Dupilumab are, on average, 1 year older.

### N3C Dupilumab analysis.

We utilized deidentified data in the National Cohort Collaborative Cohort (N3C) enclave, which currently contains 2 years of medical record data from 34 sites in the United States, to explore the association between Dupilumab use and COVID-19 outcomes. This enclave represents more than 2 million persons (including approximately 300,000 COVID-19 cases) and medical facts from more than approximately 90,000 visits. Values less than 20 have been suppressed as per current N3C publication policy.

Cohort Definitions. a) Dupilumab+: If patient had Dupilumab within 61 days prior to their first COVID-19 diagnosis date; and b) Controls+: Patients with no record of Dupilumb within 2 months prior to their first COVID-19 diagnosis date.

Outcome Definitions: a) Hospitalized: Patients who became inpatients within 6 weeks after COVID-19 diagnosis date; b) Death: If subjects’ death date is after their first COVID-19 diagnosis date; and c) With Ventilation: If patients were put on a ventilator within 6 weeks of any of their COVID-19 diagnosis dates (window is double-sided; the procedure could have been 6 weeks before or after any of their diagnosis dates).

The incidence of COVID positivity in people on Dupilumab [cohort definition 1 above / (definition 1 + 2)], along with 95% CIs, was calculated. Then, a case-control design was used. Dupilumab+ patients were matched to control+ patients in a 1:5 ratio, with exact matching on sex, race, ethnicity, N3C site, asthma, and nearest matching on age. A conditional logistic regression was used to compare COVID-19 severity outcomes within this matched subset of COVID+ patients. A sensitivity analysis was performed excluding asthma from the matching criteria.

### Virus and cell lines.

SARS-CoV-2, isolate Hong Kong/VM20001061/2020 (NR-52282), was obtained from the Biodefense and Emerging Infections Research Resources Repository (BEI Resources), National Institute of Allergy and Infectious Diseases (NIAID), National Institutes of Health (NIH). The virus was propagated in Vero C1008, Clone E6 (ATCC CRL-1586) cells cultured in Dulbecco’s Modified Eagle’s Medium (DMEM) (Gibco, Thermo Fisher Scientific, 11995040) supplemented with 10% FBS and grown at 37°C, 5% CO_2_. Initial viral stocks were used to infect Vero E6 cells, generating passage 1 (P1) stocks. These P1 stocks were then used to infect additional Vero E6 cells, generating passage 2 (P2) stocks, which were used for all experiments.

### Viral propagation.

Vero E6 cells grown to 90% confluency in T75 tissue culture flasks (Thermo Fisher Scientific) were infected with SARS-CoV-2 at a multiplicity of infection of 0.025 in serum-free DMEM. Vero E6 cells were incubated with the virus for 2 hours at 37°C, 5% CO_2_, after which the virus was removed, media was replaced with DMEM supplemented with 10% FBS, and flasks were incubated at 37°C, 5% CO_2_. After 2 days, infected flasks showed significant cytopathic effects, with more than 50% of cells unattached. Cell supernatants were collected, filtered through a 0.22μm filter (Millipore, SLGP003RS), and centrifuged at x300 *g* for ten minutes at 4°C. Cell supernatants were divided into cryogenic vials (Corning, 430487) as viral stocks and stored at –80°C until use.

### Challenge.

8–16 week-old male Tg (K18-*hACE2*) 2Prlmn (Jackson Laboratories; ref. 20) mice were challenged with 5,000 PFU of SARS-CoV-2, Hong Kong/VM20001061/2020 (BEI Resources), in 50 μL by an i.n. route under 100 μL ketamine/xylazine sedation. Mice were followed daily for clinical symptoms, which included weight loss (scores 0–5), activity (scores 0–3), fur appearance and posture (scores 0–2), and eye closure (scores 0–2). Mice were given 150 μg of anti–IL-13 (Thermo Fisher Scientific, eBio1316H; catalog 16-7135-85) or an isotype matched control IgG (Thermo Fisher Scientific, eBRG1; catalog 16-4301-85) administered on day 0, 2, and 4 after infection. For experiments utilizing hyaluronidase, 14 U in 70 μL of ovine testicular hyaluronidase (Vitrase; 200 USP U/mL) or saline control were administered i.n. following isoflurane anesthetization on day 5 after infection. For anti-CD44 experiments, 100 μg of anti-CD44 (BD Biosciences, IM7; catalog 553131) or IgG2 (BD Biosciences, A95-1; catalog 559478) were administered on day 1 after infection, and then mice were given an additional 50 μg on days 2, 3, and 4.

### IL-13 delivery in vivo.

To extend the half-life of IL-13, fusion proteins of mouse IL-13 Fc portion of IgG1 were generated (custom order with Absolute Antibody; ref. 45). Female C57BL/6 mice were anaesthetized with isoflurane inhalation and administered PBS or 10 μg IL-13 Fc i.n. (in 40 uL) on days 0 and 2. On day 3, serum and BALF was collected and stored (–80°C) until use for measurement of secreted HA levels. Lungs were inflated with 10% neutral-buffered formalin and fixed to assess histological HA deposition.

### Viral titers.

The left lobe of the lung was removed, placed in a disposable tissue grinder with 1 mL of serum-free DMEM on ice and then ground. Lung homogenates were centrifuged at x300 *g* for 10 minutes, and then the supernatants were collected and frozen at –80° C until use. Plaque assays were as described previously (20). Briefly, Vero E6 cells seeded in 12-well tissue culture plates were infected with lung homogenates serially diluted in serum-free DMEM. Plates were then incubated at 37°C, 5% CO_2_ for 2 hours to allow viral infection of the cells, before washing with sterile PBS (Gibco, Thermo Fisher Scientific, 10010-023) to remove virus and replace with an overlay of DMEM, 2.5% FBS containing 1.2% Avicel PH-101 (Sigma Aldrich). After incubation at 37°C, 5% CO_2_ for 2 days, the overlay was removed, wells were fixed with 10% formaldehyde and stained with 0.1% crystal violet to visualize plaques and calculate viral titers as PFU/mL.

### Histology.

Tissues were fixed in formaldehyde, processed, and embedded in paraffin. Slides were sectioned at 5 microns and stained with H&E (Thermo Fisher Scientific) or Periodic Acid–Schiff (Thermo Fisher Scientific; Azer) using standard protocols. Slides were scanned at x20 magnification. Histopathological scoring for lung tissue was done according to the guidelines of the American Thoracic Society (46).

For antibody and HA staining, lung sections were deparaffinized and heat-mediated antigen retrieval-performed using Tris-EDTA buffer (10 mM Tris Base [MilliporeSigma], 1 mM EDTA; 0.05 % Tween-20 [MilliporeSigma], pH 9.0; incubation 20 min 95°C). Nonspecific protein was blocked (2% donkey serum, 1% BSA, 0.05% Tween-20) prior to blocking endogenous avidin and biotin (Thermo Fisher Scientific). Lung sections were incubated with primary antibodies or HA binding protein (Supplemental Table 7) overnight at 4°C, washed in PBS containing 0.05% Tween-20 before incubation with secondary antibodies (Supplemental Table 7) for 1 hour at room temperature followed by mounting with DAPI containing fluoromount (Southern Biotech). Images were captured with an EVOS FL imaging system (Thermo Fisher Scientific). Analysis of images was performed using ImageJ software (version 2.09.0-rc69/1.52p) on sections where sample identification was blinded for the investigator. For calculation of antibody positive staining, background autofluorescence subtraction was performed to remove nonspecific staining based on a secondary-only control stain. Analysis was limited to regions of interest (airway, vessels, or parenchyma), and a threshold was then applied (by eye) to images in order to include positive staining but to exclude areas of high autofluorescence (for example red blood cells). For measurements of airway positivity, stain intensity was normalized to the length of the basement membrane. Staining for all experimental mice is shown in Supplemental Figure 6.

### Mouse bronchioalveolar lavage.

BAL fluid was collected from each animal through cannulation of the exposed trachea and flushed twice with 0.7mL of PBS. BALF samples were centrifuged at x500 *g* for 5 minutes, and supernatant was immediately frozen for later cytokine analyses. For flow cytometry, pelleted cells were resuspended in FACS (PBS + 5% FBS) buffer for staining and with Zombie NIR (BioLegend, 423105), CD45, Alexa Fluor 532 (eBioscience, 58-0459-42), CD11c, PE-Cy7 (BioLegend, 117317), CD11b, BV480 (BD Biosciences, 566117), SIGLEC F, AF700 (eBioscience, 56-1702-80), Ly-6c, FITC (BioLegend, 128005), and Ly-6G, BV650 (BioLegend, 127641) and then fixed in IC-fixation buffer (eBioscience, 00-8222-49). Samples were run on a Cytek Aurora Borealis at the University of Virginia flow cytometry core. Neutrophils are Zombie NIR−, CD45+, CD11C−, CD11B+, and Ly-6G+; eosinophils are Zombie NIR−, CD45+, CD11C−, CD11B+, and Siglec-F+; and inflammatory monocytes are Zombie NIR−, CD45+, CD11C−, CD11B+, Ly-6G−, and Ly-6C+.

Cytokine analyses were performed via Luminex Mouse 32-plex (MCYTMAG-70K-PX32, MilliporeSigma). Samples were run following manufacturers protocol after an 18-hour incubation before being run on Luminex analyzer (MAGPIX).

### RNA-Seq.

A snip from the lower inferior lobe was taken and RNA was extracted from murine lung tissue preserved in trizol by bead beating (TissueLyserII, Qiagen), followed by a phenol-chloroform extraction and RNA Isolation Kit (Qiagen). RNA quality was assessed using Agilent Tape Station RNA kit. Library preparation, sequencing, quality control, and read mapping were performed by the Genome Analysis and Technology Core, RRID:SCR_018883. Briefly, ribosomal RNA was depleted using the rRNA depletion kit (NEB E6310). Following rRNA depletion, cDNA libraries were prepared using the NEB ultra-directional library preparation kit 2.0 (NEB, E7760) and indexed using the NEBNext Multiplex Oligos for Illumina (NEB, E7335, E7500). Library size and purity were verified using the Agilent Tape Station D5000 HS kit. Library concentration was measured with a qubit DNA HS assay (Invitrogen, Thermo Fisher Scientific). Libraries concentrations were normalized, and 15 libraries were multiplexed per run. Diluted libraries were sequenced on the Illumina Nextseq 500 using a 150 high output kit (400 million reads, 2×75 bp paired end, 150 cycles). 

### RNA-Seq data analysis.

RNA-Seq reads were first processed using Cutadapt (47) to trim the adapter sequences and then the quality of the reads was assessed by FastQC (https://www.bioinformatics.babraham.ac.uk/projects/fastqc/) (48) and MultiQC (49). After these processes, the reads were aligned to the mouse Ensembl GRCh38.76 primary assembly using STAR v2.5.3a (50) in a 2-passing mode to generate a gene matrix for differential gene expression. Differentially expressed genes were determined using the DESeq2 package (51) in Rstudio (http://www.rstudio.com/).

Enrichment analysis was applied to the total gene counts using the reactome GSA package (52) in Rstudio. Enrichment analysis was applied to the total gene counts using the CAMERA algorithm (53).

### Data availability.

All corresponding data and code are available through Dryad or NPG (https://www.ncbi.nlm.nih.gov/geo/info/linking.html; GSE180557).

### Statistics.

For the clinical study, a total of 47 cytokines, chemokines, and growth factors were measured in 178 patients who were positive with COVID-19 with plasma samples. For cytokines of interest, levels at the time of COVID-19 diagnosis or admission were compared between patients with different severities of illness using a Mann-Whitney U test. The pheatmap function in the pheatmap library in R (R-project.org) was used for hierarchical clustering. Cytokines were scaled by row (patients), and the clustering was calculated using the complete linkage method. Statistical analyses were performed using GraphPad Prism and R.

Since these cytokine measurements were highly correlated, PCA was performed to identify distinct features among correlated cytokines, and thus reduce the dimensionality. Due to their skewed distributions and variable scales, the cytokines were first log-transformed and then standardized with a mean of 0 and standard deviation of 1. The first 5 principal components (PCs) captured 62% of total variation in the cytokines. For each PC, those cytokines with a loading score of 0.5 or above were retained, showing the strength of their influences within the component. Additionally, the network analysis using the qgraph package in R was performed to characterize the complex structural relationships among cytokine measurements and optimized with graphical LASSO. The nodes represented individual cytokines, and the edges represented their correlations in that highly correlated cytokines were connected closer with thick edges (https://cran.rproject.org/web/packages/qgraph/qgraph.pdf).

Survival Analysis was performed for patients from the time to ventilation since symptom onset. Those who were not ventilated were censored at 40 days. Patients were classified into 4 quartiles based on the cumulative distribution of IL-13 levels, and the probabilities of ventilation were estimated by the Kaplan-Meier method. Since the 2 upper quartiles had statistically identical results in the preliminary analysis, they were combined in the final log-rank test and Cox regression done for their relative performance over the lower quartiles, and were corrected for sex, age, and cumulative number of comorbidities (including, diabetes, cancer, stroke, and heart, liver, kidney, or lung diseases).

An ROC curve was generated using pROC library in RStudio for IL-13 alone and in combination with IL-6, IL-8, and MIP-1β, which were selected as within the top predictors for ventilation identified by conditional random forests analysis (data not shown).

For clinical scores, weight loss, and scored histological sections of mouse studies, a 2-tailed Student’s *t* test was used to determine statistical significance. For comparison between 3 groups, Tukey’s honestly significant difference (HSD) test was used. For IHC sections with multiple quantified images per mouse or human tissue, response differences between groups (e.g., infected versus uninfected or IgG versus aIL-13) were evaluated in the mixed-effects model to account for within-individual correlation, and distributions were log-transformed where appropriate. *P* value < 0.05 was considered significant.

### Study design.

Discarded human plasma samples from COVID-19 positive and negative patients at the University of Virginia Medical Center were collected for cytokine and growth factor analyses. The collection of biological specimens and de-identified patient information was approved by the University of Virginia Institutional Review Board (IRB-HSR 22231 and 200110). In mice, neutralizing antibodies or isotype controls were used to assess the role of IL-13 during COVID-19. All mouse work was approved by the University of Virginia Institutional Animal Care and Use Committee, and all procedures were performed in the University certified animal Biosafety Level Three laboratory.

## Author contributions

AND, TES, BJM, AJD, and WAP contributed in conceptualization; SLB, MMA, GBM, and BJM contributed in methodology; JRD, GAB, and MGS contributed in validation; TES, CM, SP, RP, BTB, JMS, JZM, and GRL contributed in formal analysis; AND, MDP, AJM, and BTB contributed resources; MKY and RMC contributed in data curation; AND contributed in writing of the original draft; all authors contributed in review and editing; AND contributed in visualization; WAP and JEA contributed in supervision; WAP, JEA, RP, SP, JMS, and BTB contributed in funding acquisition; and WAP contributed in project administration. 

## Supplementary Material

Supplemental data

## Figures and Tables

**Table 1:**
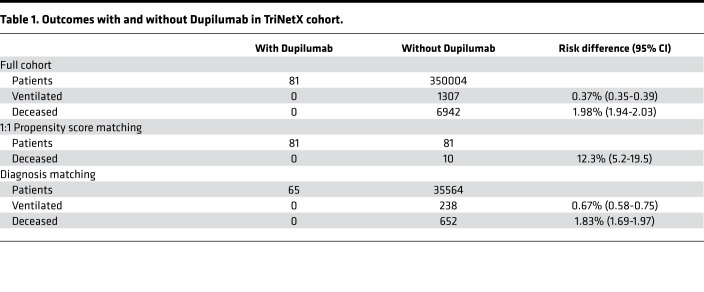
Outcomes with and without Dupilumab in TriNetX cohort.

**Table 2:**
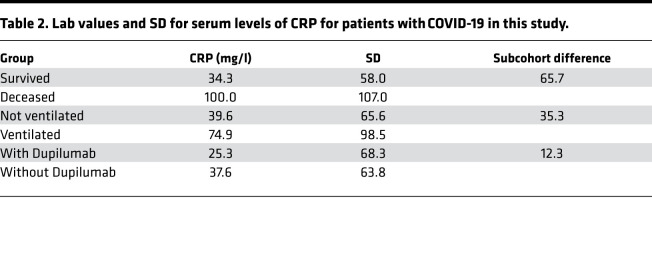
Lab values and SD for serum levels of CRP for patients with COVID-19 in this study.

**Table 3:**
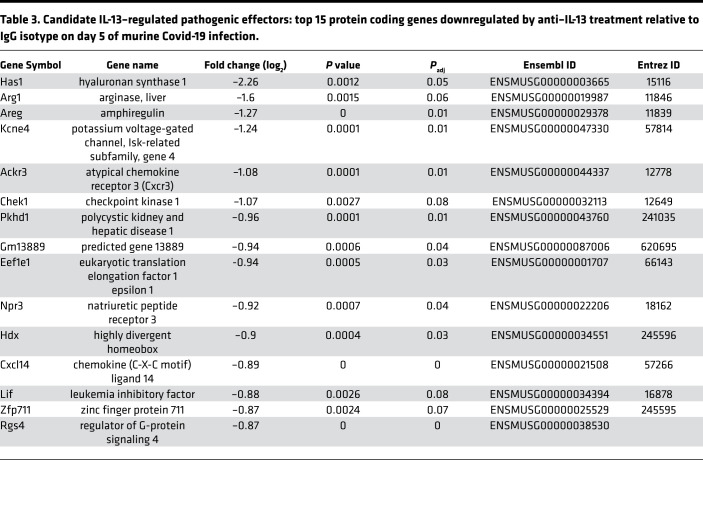
Candidate IL-13–regulated pathogenic effectors: top 15 protein coding genes downregulated by anti–IL-13 treatment relative to IgG isotype on day 5 of murine Covid-19 infection.

## Version 1 06/29/2021

In-Press Preview

## Version 2 08/09/2021

Electronic publication
