# Supplementary material for: IL-13 is a driver of COVID-19 severity
Source: JCI Insight. 2021 Aug 9;6(15):e150107. doi: 10.1172/jci.insight.150107 (PMC8410056; doi:10.1172/jci.insight.150107)
Supplement: Supplemental data [file jciinsight-6-150107-s114.pdf]

A

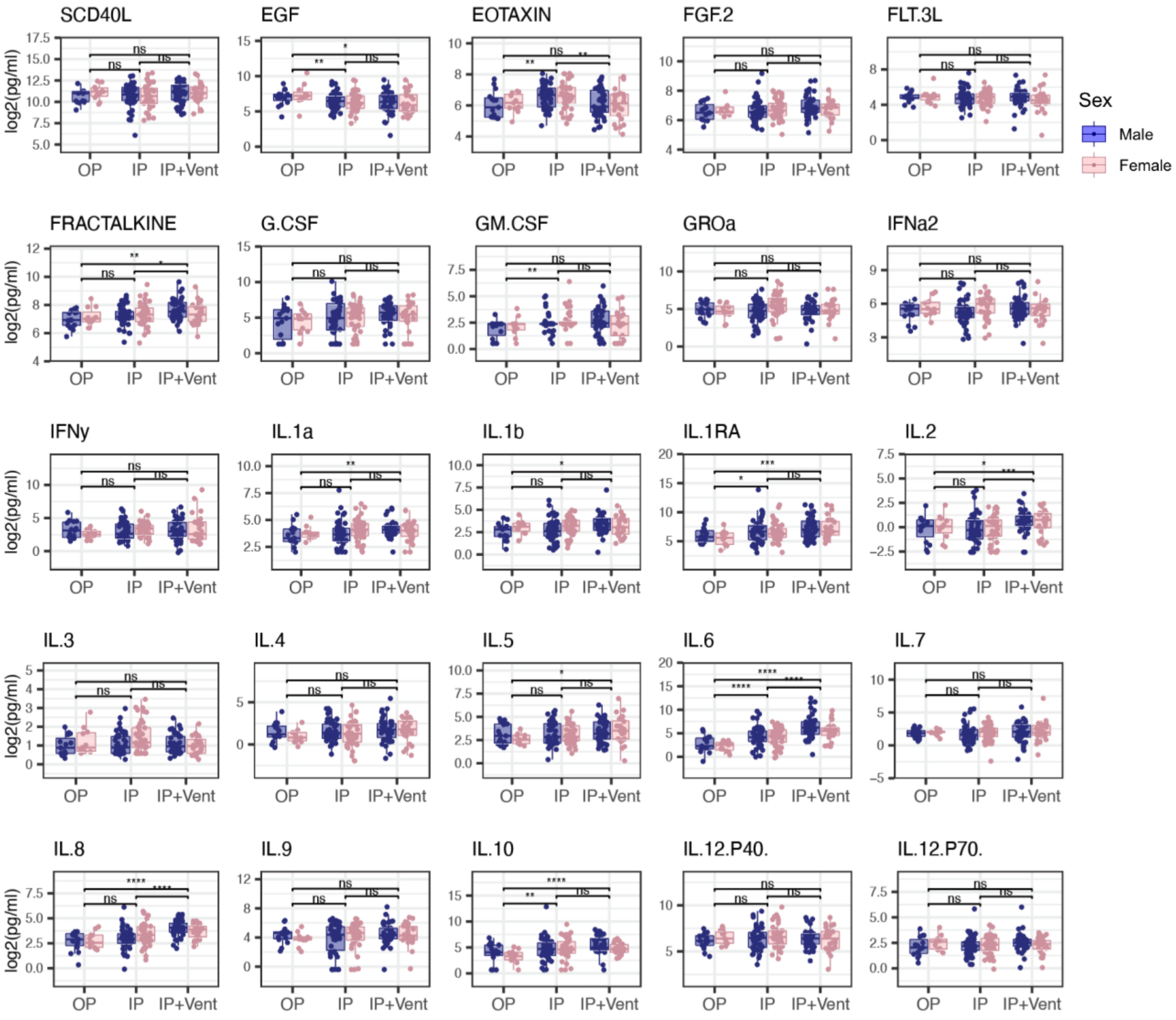

SCD40L
EGF
EOTAXIN
FGF.2
FLT.3L
FRACTALKINE
G.CSF
GM.CSF
GROa
IFNa2
IFNy
IL.1a
IL.1b
IL.1RA
IL.2
IL.3
IL.4
IL.5
IL.6
IL.7
IL.8
IL.9
IL.10
IL.12.P40.
IL.12.P70.
log2(pg/ml)
OP
IP
IP+Vent
Sex
Male
Female

B

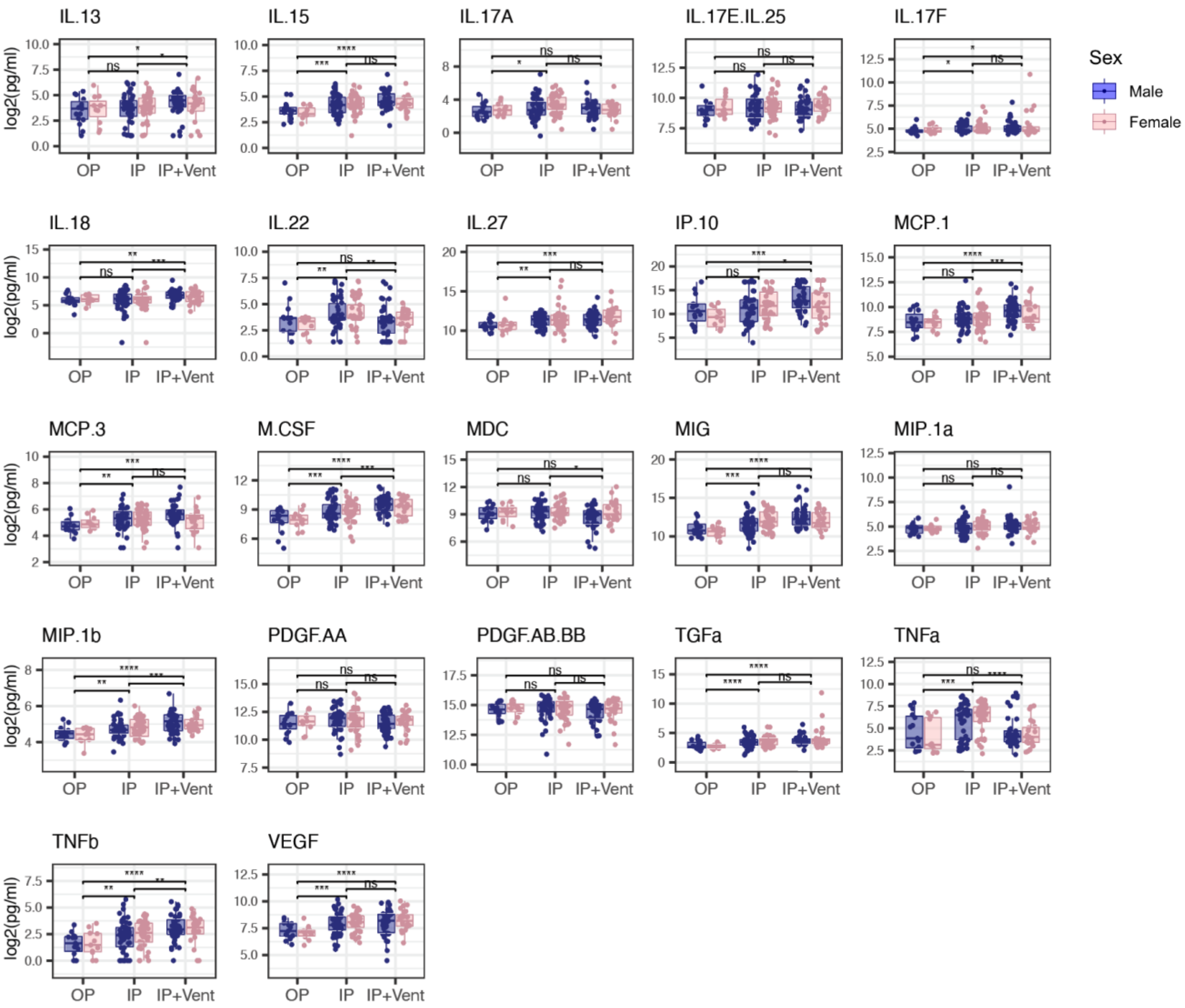
IL.13
IL.15
IL.17A
IL.17E.IL.25
IL.17F
IL.18
IL.22
IL.27
IP.10
MCP.1
MCP.3
M.CSF
MDC
MIG
MIP.1a
MIP.1b
PDGF.AA
PDGF.AB.BB
TGFa
TNFa
TNFb
VEGF
log2(pg/ml)
OP
IP
IP+Vent
Sex
Male
Female

C

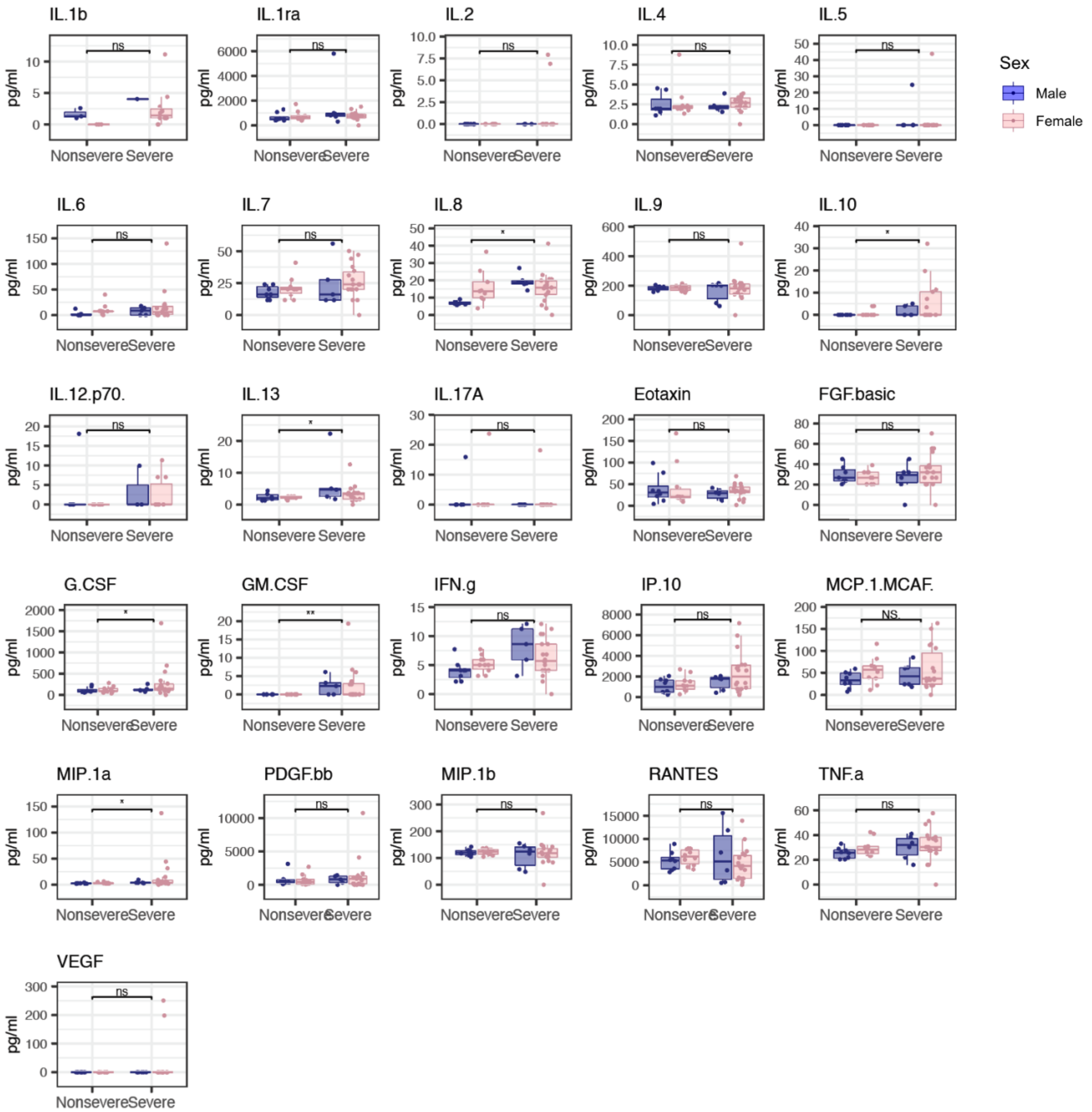
IL.1b
IL.1ra
IL.2
IL.4
IL.5
IL.6
IL.7
IL.8
IL.9
IL.10
IL.12.p70.
IL.13
IL.17A
Eotaxin
FGF.basic
G.CSF
GM.CSF
IFN.g
IP.10
MCP.1.MCAF.
MIP.1a
PDGF.bb
MIP.1b
RANTES
TNF.a
VEGF
pg/ml
Nonsevere
Severe
ns
*
**
NS.
Sex
Male
Female

Figure S1. Visualization of cytokines in outpatients and inpatients with COVID-19 and in uninfected controls. A) A total of 47 cytokines, chemokines and growth factors were measured from plasma of COVID-19 positive patients (N=152), and cytokines of interest were plotted by sex (blue, Male; pink, Female) and compared between patients with differing severity of illness using a Mann-Whitney U test. (OP, outpatient; IP, inpatient; IP+Vent, ventilated inpatient). B) 26 cytokines and growth factors were measured in plasma from 19 non-severe and 26 severe (requiring supplemental oxygen) COVID patients from VCU. Nonsevere and severe groups for each cytokine were compared using a Mann-Whitney U test.

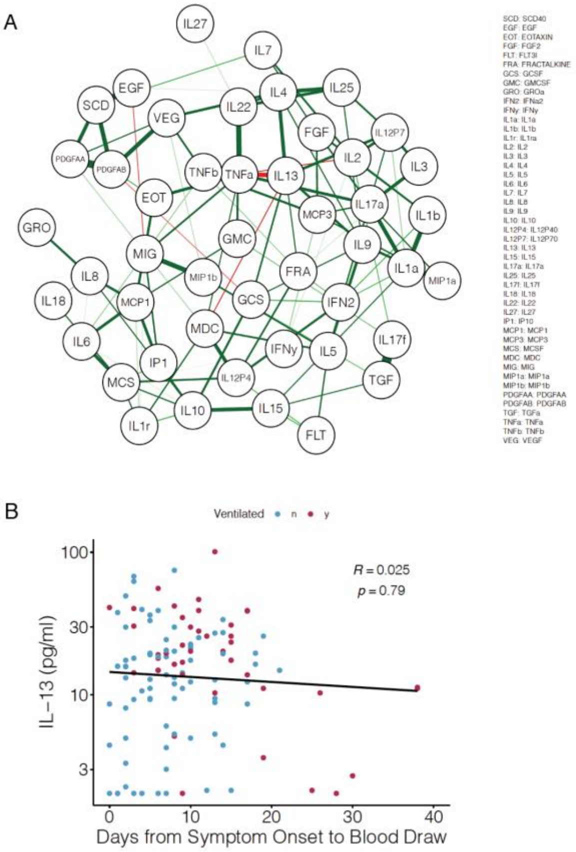
A
SCD: SCD40
EGF: EGF
EOT: EOTAXIN
FGF: FGF2
FLT: FLT3l
FRA: FRACTALKINE
GCS: GCSF
GMC: GMCSF
GRO: GROa
IFN2: IFNa2
IFNy: IFNy
IL1a: IL1a
IL1b: IL1b
IL1r: IL1ra
IL2: IL2
IL3: IL3
IL4: IL4
IL5: IL5
IL6: IL6
IL7: IL7
IL8: IL8
IL9: IL9
IL10: IL10
IL12P4: IL12P40
IL12P7: IL12P70
IL13: IL13
IL15: IL15
IL17a: IL17a
IL25: IL25
IL17f: IL17f
IL18: IL18
IL22: IL22
IL27: IL27
IP1: IP10
MCP1: MCP1
MCP3: MCP3
MCS: MCSF
MDC: MDC
MIG: MIG
MIP1a: MIP1a
MIP1b: MIP1b
PDGFAA: PDGFAA
PDGFAB: PDGFAB
TGF: TGFa
TNFa: TNFa
TNFb: TNFb
VEG: VEGF
B
Ventilated n y
R = 0.025
p = 0.79
IL−13 (pg/ml)
Days from Symptom Onset to Blood Draw

Figure S2. Network analysis of cytokine genes, and relationship of IL-13 with days from symptom onset in patients with COVID-19. A) The network analysis captured the structural relationships among cytokine measurements with graphical LASSO. The nodes represented individual cytokines and edges represented their correlations in that highly correlated cytokines were connected closer with thick edges. Green line = positive correlation; red line = negative correlation. B) IL-13 plasma levels and corresponding days from symptom onset to time of blood draw were plotted (N= 125). Linear relationship was evaluated with spearman linear regression. R-squared and p-values shown. Red = required mechanical ventilation; blue = did not require mechanical ventilation.

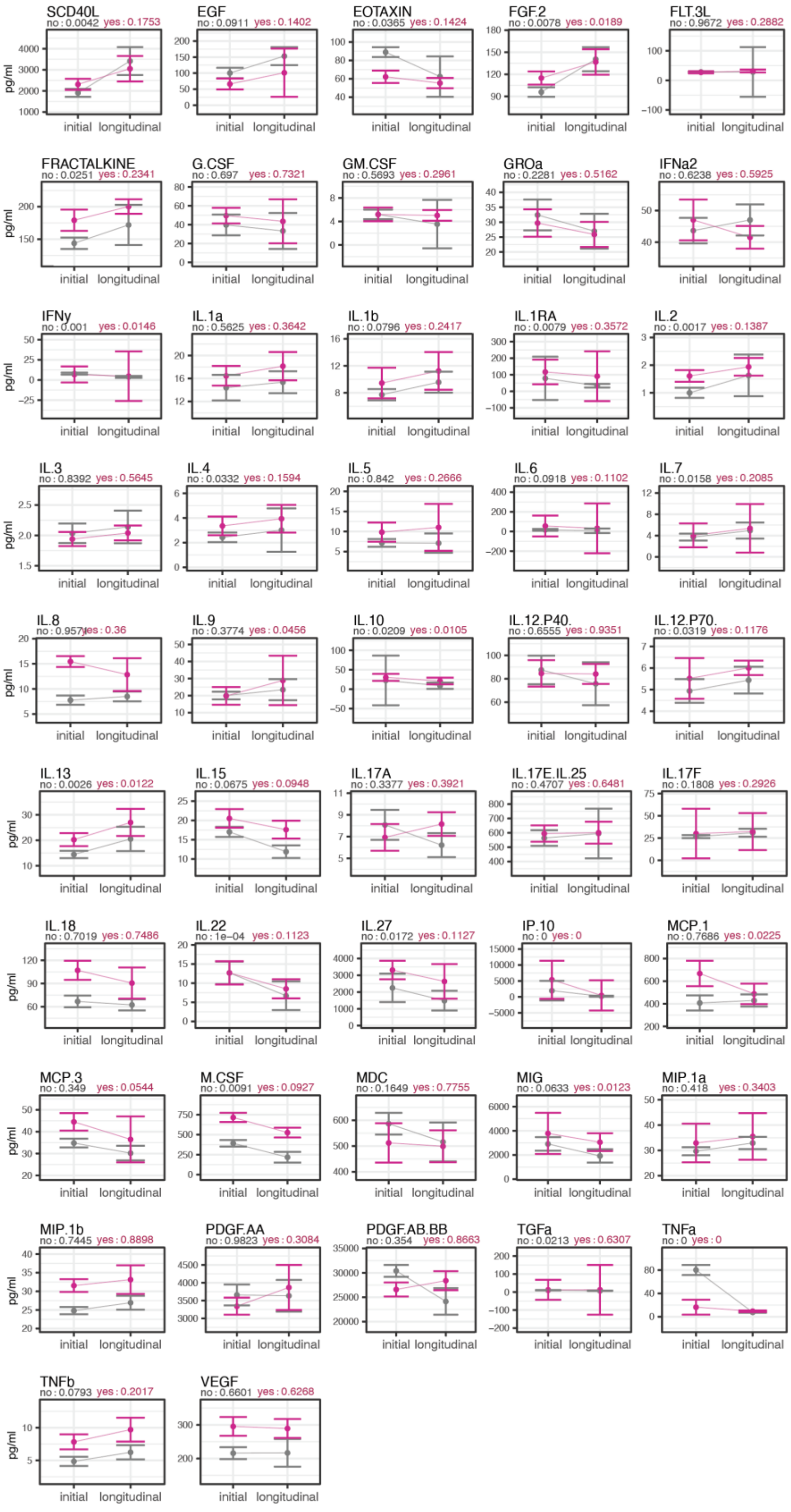
SCD40L no : 0.0042 yes : 0.1753
EGF no : 0.0911 yes : 0.1402
EOTAXIN no : 0.0365 yes : 0.1424
FGF.2 no : 0.0078 yes : 0.0189
FLT.3L no : 0.9672 yes : 0.2882
FRACTALKINE no : 0.0251 yes : 0.2341
G.CSF no : 0.697 yes : 0.7321
GM.CSF no : 0.5693 yes : 0.2961
GROa no : 0.2281 yes : 0.5162
IFNa2 no : 0.6238 yes : 0.5925
IFNy no : 0.001 yes : 0.0146
IL.1a no : 0.5625 yes : 0.3642
IL.1b no : 0.0796 yes : 0.2417
IL.1RA no : 0.0079 yes : 0.3572
IL.2 no : 0.0017 yes : 0.1387
IL.3 no : 0.8392 yes : 0.5645
IL.4 no : 0.0332 yes : 0.1594
IL.5 no : 0.842 yes : 0.2666
IL.6 no : 0.0918 yes : 0.1102
IL.7 no : 0.0158 yes : 0.2085
IL.8 no : 0.957 yes : 0.36
IL.9 no : 0.3774 yes : 0.0456
IL.10 no : 0.0209 yes : 0.0105
IL.12.P40. no : 0.6555 yes : 0.9351
IL.12.P70. no : 0.0319 yes : 0.1176
IL.13 no : 0.0026 yes : 0.0122
IL.15 no : 0.0675 yes : 0.0948
IL.17A no : 0.3377 yes : 0.3921
IL.17E.IL.25 no : 0.4707 yes : 0.6481
IL.17F no : 0.1808 yes : 0.2926
IL.18 no : 0.7019 yes : 0.7486
IL.22 no : 1e−04 yes : 0.1123
IL.27 no : 0.0172 yes : 0.1127
IP.10 no : 0 yes : 0
MCP.1 no : 0.7686 yes : 0.0225
MCP.3 no : 0.349 yes : 0.0544
M.CSF no : 0.0091 yes : 0.0927
MDC no : 0.1649 yes : 0.7755
MIG no : 0.0633 yes : 0.0123
MIP.1a no : 0.418 yes : 0.3403
MIP.1b no : 0.7445 yes : 0.8898
PDGF.AA no : 0.9823 yes : 0.3084
PDGF.AB.BB no : 0.354 yes : 0.8663
TGFa no : 0.0213 yes : 0.6307
TNFa no : 0 yes : 0
TNFb no : 0.0793 yes : 0.2017
VEGF no : 0.6601 yes : 0.6268
pg/ml
initial
longitudinal

Figure S3. Initial vs longitudinal comparison for cytokines in COVID-19 positive patients. 47 cytokines, chemokines and growth factors measured in plasma samples from COVID-19 positive patients at the UVA Medical center. Mean and SE plotted for patients who were (pink) or were not (grey) ventilated at initial and longitudinal sample collection. Due to incomplete overlap in available samples between initial (N = 183) and longitudinal (N = 70) blood draws, Mann-Whitney U test was performed comparing initial to longitudinal groupings.

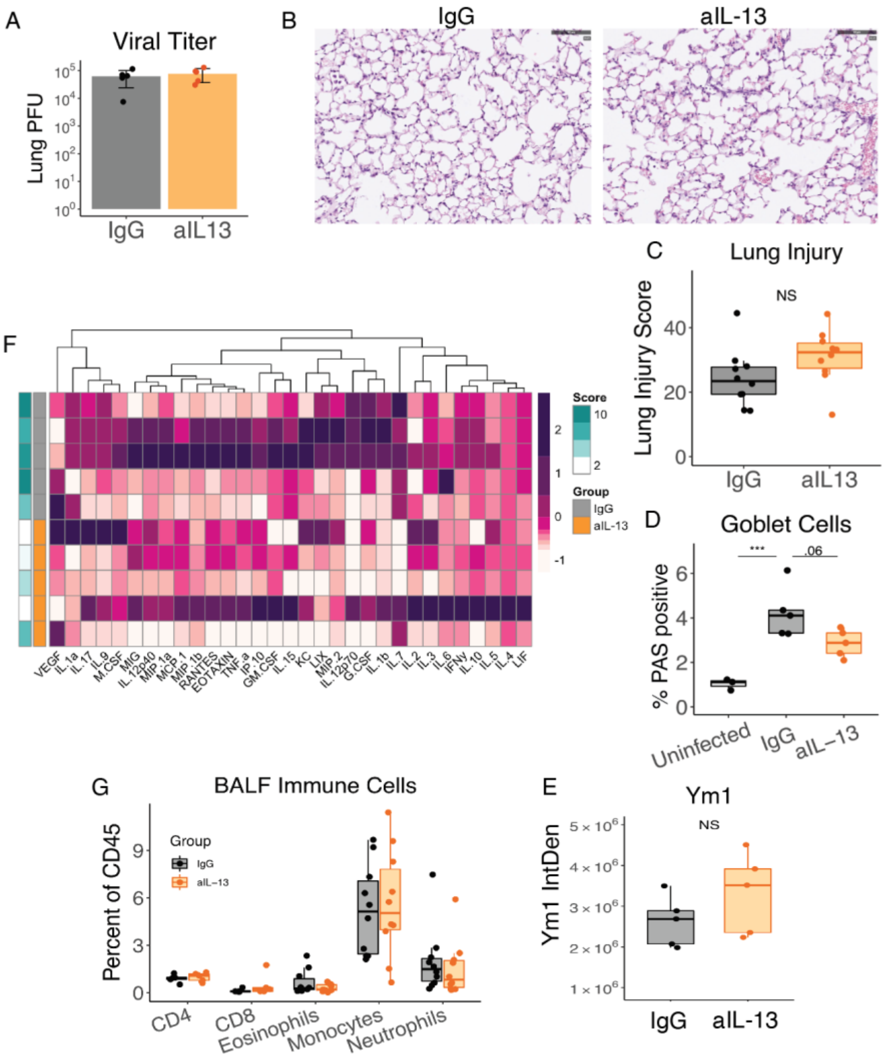
A
Viral Titer
Lung PFU
IgG
aIL13
B
IgG
aIL-13
C
Lung Injury
Lung Injury Score
NS
IgG
aIL13
D
Goblet Cells
% PAS positive
***
.06
Uninfected
IgG
aIL−13
E
Ym1
Ym1 IntDen
NS
IgG
aIL-13
F
Score
Group
IgG
aIL-13
VEGF
IL.1a
IL.17
IL.9
M.CSF
MIG
IL.12p40
MIP.1a
MCP.1
MIP.1b
RANTES
EOTAXIN
TNF.a
IP.10
GM.CSF
IL.15
KC
LIX
MIP.2
IL.12p70
G.CSF
IL.1b
IL.7
IL.2
IL.3
IL.6
IFNy
IL.10
IL.5
IL.4
LIF
G
BALF Immune Cells
Percent of CD45
Group
IgG
aIL−13
CD4
CD8
Eosinophils
Monocytes
Neutrophils

Figure S4. Impact of anti-IL-13 on lung injury and inflammation in a mouse model of COVID-19. Mice were infected with $5x10^3$ PFU of SARS-CoV-2 on day 0 and given 150 µg of anti-IL-13 or IgG isotype control on days 0, 2, and 4. On day five, mice were euthanized and bronchoalveolar lavage (BAL) fluid collected. For histology, lungs were inflated with formalin before removing and fixing prior to H&E staining. A) Viral burden in lungs on day five pi was measured by plaque forming units (PFU) (N = 5 mice/group; 2 repeats; t-test). B) Hematoxylin and eosin stain of infected mouse lung with or without anti-IL-13 and C) Quantified lung injury score. (N = 10 mice/group; t-test) D) Goblet cells quantified from PAS staining of lung tissue from day five p.i. (N = 5 mice/group; Tukey's HSD) E) Ym1 with or without anti-IL-13. (N = 5 mice/group; mixed effect model) F) Cytokines in BAL were measured by Luminex (plotted with group identity and clinical score) (N = 5 mice/group) and G) immune cells in BAL quantified by flow cytometry. (N = 10 mice/group; t-test). NS= not significant; *** = p < 0.01

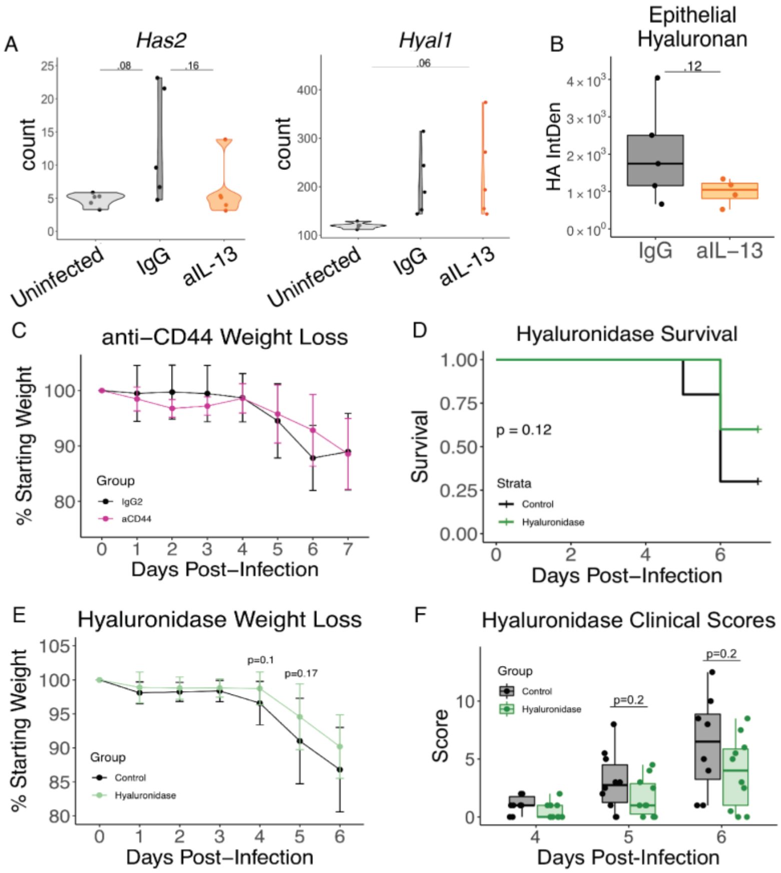
A
Has2
count
.08
.16
Uninfected
IgG
aIL-13
Hyal1
count
.06
Uninfected
IgG
aIL-13
B
Epithelial Hyaluronan
HA IntDen
.12
IgG
aIL-13
C
anti−CD44 Weight Loss
% Starting Weight
Group
IgG2
aCD44
Days Post−Infection
D
Hyaluronidase Survival
Survival
p = 0.12
Strata
Control
Hyaluronidase
Days Post−Infection
E
Hyaluronidase Weight Loss
% Starting Weight
p=0.1
p=0.17
Group
Control
Hyaluronidase
Days Post−Infection
F
Hyaluronidase Clinical Scores
Score
Group
Control
Hyaluronidase
p=0.2
p=0.2
Days Post-Infection

Figure S5. Hyaluronan and its receptor contribute driving severe COVID-19 in mice. Mice were infected with $5x10^3$ PFU of SARS-CoV-2 on day 0 and given 150 µg of anti-IL-13 or IgG isotype control on days 0, 2, and 4. On day five, mice were euthanized and sections of lung were stored in trizol. RNAseq was done on lung tissue. Read counts of A) hyaluronan synthase 2 (*Has2*) and hyaluronidases 1 (*Hyal1*) were analyzed between anti-IL-13 treated mice and isotype controls. (N = 5 mice/group; Tukey's HSD). B) Quantification of intensity of epithelial hyaluronan from fluorescent staining (log-transformed, mixed-model). Infected mice were administered anti-CD44 antibodies or isotype IgG2 control on days 1,2,3 and 4 pi C) weight loss were quantified; combined two, independent experiments (N = 10 mice/group; t-test). D) Kaplan-Meier survival curve (log rank), E) weight loss and F) clinical scores from mice infected with $5x10^3$ PFU of SARS-CoV-2 who were administered hyaluronidase on day five pi; combined, two independent experiments (N = 5 mice/group; E,F: t-test). *=p<0.05; **=p<0.005

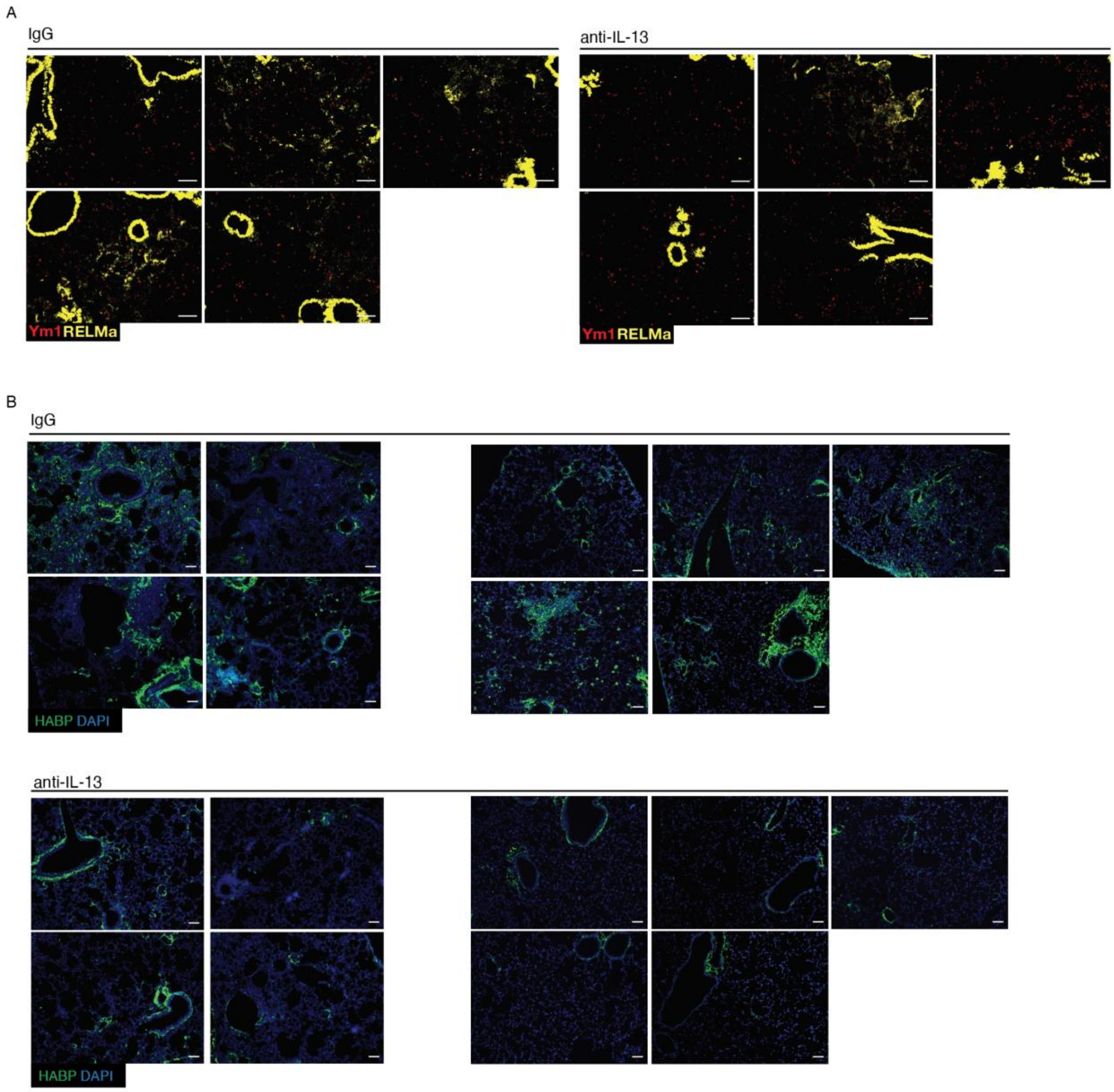
A
IgG
anti-IL-13
Ym1 RELMa
Ym1 RELMa
B
IgG
HABP DAPI
anti-IL-13
HABP DAPI

5

Figure S6. Staining of Ym1, RELMα and HA for all experimental mice. (Related to Figure 3 and 4). Mice received i.p. injections of anti-IL-13 on days 0 and 2 pi, were euthanized on day 5. A) Lung sections stained with Ym1 (red) and RELMα (yellow) for SARS-CoV-2 infected mice treated with IgG or anti-IL-13. Each image is representative of an individual mouse, scale bar = 70μm. B) Lung sections stained with hyaluronan binding protein (HABP, green) and DAPI nuclei stain (blue) for SARS-CoV-2 infected mice treated with IgG or anti-IL-13. Each image is representative of an individual mouse, scale bar = 70μm

Table S1. Age, sex and clinical status of UVA patients.

	Outpatient (n = 26)	Inpatient (n = 152)
**Age**	42.4 (15.1)	59.2 (16.7)
**Sex**		
• **Male**	11 (42.3%)	66 (43.3%)
• **Female**	15 (57.7%)	86 (56.6%)
**Race**		
• **Caucasian**	5 (19.2%)	60 (39.5%)
• **African-American**	7 (26.9%)	34 (22.4%)
• **Asian**	2 (7.7%)	3 (2.0%)
• **Other**	12 (46.1%)	55 (36.2%)
**Ethnicity**		
• **Hispanic**	13 (50.0%	61 (40.1%)
• **Non-Hispanic**	12 (50.0%)	91 (59.9%)
**Timing of blood draw from day of symptom onset (mean, SD)**	4.40 (2.95)	11.3 (11.2)
**Missing/unknown**	6 (23.1%)	40 (26.3%)
**Respiratory Status at time of blood draw**		
• **No oxygen requirement**	25 (96.2%)	45 (29.6%)
• **Supplemental oxygen only**	1 (3.8%)	57 (37.5%)
• **Mechanical ventilation**	0	50 (32.9%)
**At any time during illness**		
• **No oxygen requirement**	25 (96.2%)	28 (18.4%)
• **Supplemental oxygen only**	1 (3.8%)	59 (38.8%)
• **Mechanical ventilation**	0	65 (42.8%)
**Ct Value (mean, SD)**	28.2 (9.22)	27.1 (6.15)
**Missing/Unknown**	11 (42.3%)	113 (74.3%)
**Comorbidity***	7 (26.9%)	101 (66.4%)
• **Diabetes**	5 (19.2%)	66 (43.4%)
• **Cancer**	1 (3.8%)	14 (9.2%)
• **Immunosuppression**	0	11 (7.2%)
• **Kidney Disease**	0	30 (19.7%)
• **Heart Disease**	1 (3.8%)	28 (18.4%)
• **Lung Disease**	1 (3.8%)	22 (14.5%)
• **Liver Disease**	0	2 (1.3%)
• **Stroke**	1 (3.8%)	13 (8.6%)
*Presence of any one of these pre-existing illnesses; diabetes, cancer, kinder, heart, lung, or liver disease, stroke, organ transplant, other immunosuppression. (William, E.J. et al, OpenSAFELY: factors associated with COVID-19 death in 17 million patients. Nature https://doi.org/10.1038/s41586-020-2521-4)		

Table S2. Contributors to Component 1 in PCA plot. Principal component analysis was performed using the Proc Factor in SAS. For principal component one, those cytokines with a loading score of 0.5 or above were retained.

Order	Cytokine	PC1 Loading
**1**	IL-1a	0.86636
**2**	MCP-3	0.82474
**3**	IL-1b	0.79245
**4**	Il-12p70	0.79093
**5**	MIP-1a	0.78886
**6**	IL-13	0.77694
**7**	IL-2	0.76816
**8**	IL-9	0.75838
**9**	FGF2	0.75743
**10**	IL-17a	0.73832
**11**	Fractalkine	0.72882
**12**	IFNa2	0.71946
**13**	IL-25	0.71022
**14**	TNF-b	0.70035
**15**	IL-15	0.67703

Table S3. Age, sex and clinical status of VCU patients.

	Non-severe (n = 22)	Severe (n = 25)
**Age**	48.3 (17.0)	30.2 (9.3)
**Sex**		
• **Male**	10 (45.5%)	18 (72.0%)
• **Female**	12 (54.5%)	7 (28.0%)
**Race**		
• **Caucasian**	1 (4.5%)	5 (20.0%)
• **African-American**	17 (77.3%)	19 (76.0%)
• **Asian**	1 (4.5%)	0 (0.0%)
• **Other**	3 (13.6%)	1 (4.0%)
**Ethnicity**		
• **Hispanic**	3 (13.6%)	1 (4.0%)
• **Non-Hispanic**	19 (86.4%)	24 (96.0%)
**Timing of blood draw from day of symptom onset (if known) (mean, SD)**	6.95 (3.43)	10.1 (12.3)
**Respiratory Status at time of blood draw**		
• **No oxygen requirement**	15 (68.2%)	2 (8.0%)
• **Supplemental oxygen only**	7 (31.8%)	20 (80.0%)
• **Mechanical ventilation**	0 (0.0%)	3 (12.0%)
**At any time during illness**		
• **No oxygen requirement**	15 (68.2%)	2 (8.0%)
• **Supplemental oxygen only**	7. (31.8%)	15 (60.0%)
• **Mechanical ventilation**	0 (0.0%)	8 (32.0%)
**Comorbidity***	15 (68.2%)	21 (84%)
• **Diabetes**	9 (40.9%)	10 (40.0%)
• **Cancer**	3 (13.6%)	6 (24.0%)
• **Immunosuppression**	4 (18.2%)	2 (8.0%)
• **Kidney Disease**	4 (18.2%)	5 (20.0%)
• **Heart Disease**	6 (27.3%)	6 (24.0%)
• **Lung Disease**	6 (27.3%)	6 (24.0%)
• **Liver Disease**	2 (9.1%)	4 (16.0%)
• **Stroke**	1 (4.5%)	2 (8.0%)
*Presence of any one of these pre-existing illnesses; diabetes, cancer, kinder, heart, lung, or liver disease, stroke, organ transplant, other immunosuppression. (William, E.J. et al, OpenSAFELY: factors associated with COVID-19 death in 17 million patients. Nature https://doi.org/10.1038/s41586-020-2521-4)		

Table S4. Enriched pathways in differentially regulated genes in murine COVID-19 infection. Enrichment analysis was applied to the total gene counts using the CAMERA algorithm. Pathways are arranged by descending number of entities found.

**Pathway Name**	Entities Found	Entities Total	Entities Ratio	Entities Pval	Entities FDR	Rxns Found	Rx ns Total	Rxns Ratio	RNA seq
Signaling by Interleukins	581	647	0.044	1.54E-03	2.91E-02	489	493	0.038	▲
Deubiquitination	259	288	0.02	1.25E-03	2.43E-02	77	77	0.006	▲
**Interleukin-4 and Interleukin-13 signaling**	**187**	**216**	**0.015**	**1.68E-03**	**3.12E-02**	**46**	**47**	**0.004**	▲
Antiviral mechanism by IFN-stimulated genes	81	94	0.006	1.29E-03	2.5E-02	27	31	0.002	▲
FCGR3A-mediated IL10 synthesis	45	141	0.01	1.38E-03	2.66E-02	20	20	0.002	▼
Aquaporin-mediated transport	44	68	0.005	1.72E-03	3.18E-02	24	25	0.002	▼
Antimicrobial peptides	40	123	0.008	1.61E-03	3.02E-02	39	58	0.004	▲
Glucagon signaling in metabolic regulation	32	40	0.003	1.81E-03	3.29E-02	6	6	0	▼
Interaction between L1 and Ankyrins	28	33	0.002	1.14E-03	2.25E-02	4	4	0	▼
Dissolution of Fibrin Clot	12	14	0.001	1.79E-03	3.27E-02	19	19	0.001	▲
Defective HLCS causes multiple carboxylase deficiency	6	10	0.001	1.48E-03	2.81E-02	4	4	0	▼

Table S5. Patient characteristics for the full cohort with and without usage of Dupilumab.

Characteristics	With Dupilumab	Without Dupilumab
**Age**		
Age at index event (yrs ± 1σ std dev)	44.3 ± 17.7	43.3 ± 20.9
**Sex**		
Female	68%	55%
Male	32%	45%
**Ethnicity**		
Race: White	59%	53%
Race: Black	24%	15%
ICD 10 R00-R99 ("Symptoms, signs and abnormal clinical and laboratory findings, not elsewhere classified")	91%	59%
**Vitals**		
Respiratory rate [breaths/min]	16.6 ± 6.5	17.0 ± 29.8
Heart rate [beats/min]	81.3 ± 12.1	81.6 ± 17.8
Oxygen saturation [%]	88.4 ± 21.1	85.6 ± 23.1
BMI [kg/m^2]	33 ± 8.4	29.5 ± 8.4
Blood pressure [mm Hg]	128 / 77	127 / 76
**Procedures performed**		
Medicine Services and Procedures	70%	41%
Evaluation and Management Services	75%	51%
Medical and Surgical Procedures	21%	17%
Anesthesia	19%	8%
**Medications** (co-medications taken)		
Dermatological agents	96%	50%
Musculoskeletal medications	65%	39%
Otics agents (infections of the ear)	23%	13%
Hormones	95%	44%
Respiratory agents	89%	44%
CNS-acting agents	86%	53%
Antimicrobials	86%	47%
Ophthalmic agents	84%	47%
Gastrointestinal medications	81%	42%
Cardiovascular medications	75%	39%
Genitourinary medications	70%	34%
**Lab-measured properties**		
**Metabolic panel**		
Sodium [mmol/L]	139 ± 2.44	139 ± 3.16
Potassium [mmol/L]	4.07 ± 0.38	4.13 ± 0.54
Chloride [mmol/L]	103 ± 3.66	103 ± 4.14
Bicarbonate [mmol/L]	24.6 ± 2.58	25.4 ± 3.18

Urea nitrogen [mg/dL]	13.5 ± 6.65	15.6 ± 10.2
Creatinine [mg/dL]	0.834 ± 0.226	1.01 ± 2.07
Glucose [mg/dL]	124 ± 63.5	114 ± 54.9
Calcium [mmol/L]	9.3 ± 0.527	9.26 ± 0.582
Magnesium [mmol/L]	1.98 ± 0.3	2 ± 0.426
Phosphate [mmol/L]	3.3 ± 0.604	3.49 ± 1.08
**Complete Blood count**		
Erythrocytes [Mill/µL]	4.6 ± 0.521	4.5 ± 0.669
Leukocytes [1000/µL]	8.64 ± 2.66	8.33 ± 4.36
Hemoglobin [g/dL]	13.3 ± 1.8	13.2 ± 2.01
Hematocrit [%]	39.3 ± 8.39	39.4 ± 6.80
**Liver function**		
ALT [U/l]	25.6 ± 17.9	29 ± 48.8
AST [U/l]	25.2 ± 16.0	28.1 ± 71.8
**Coagulation**		
INR	1.02 ± 0.38	1.17 ± 0.74
**Lipid panel**		
Cholesterol in LDL [mg/dL]	102 ± 30.6	101 ± 35.8
Cholesterol in HDL [mg/dL]	54.1 ± 14.0	51 ± 16.2
Triglycerides [mg/dL]	127 ± 71.3	131 ± 109

Table S6. N3C Dupilumab COVID severity outcomes. A total of 785 patients with a record of dupilumab prescription were in the N3C Data Enclave on November 16, 2020. Of these, 31 Dupilumab patients had a COVID+ test within 61 days after a dupilumab dose, resulting in a test positivity rate of 3.9% (95% CI: 2.8, 5.6). A total of 247,391 COVID+ patients with no record of Dupilumab were available for selection of matched controls. Five matches could be found for each patient. In the matched analytic dataset of COVID+ patients, no differences were seen in the hospitalization (OR=0.64, p=0.57) or death rates (p>0.99); though <20 deaths were seen in the entire dataset. In hospitalized patients, no differences were observed in the rates of ECMO (p>0.99) or IMV (p>0.99).

	With concurrent Dupilumab	Matched controls without Dupilumab	Odds ratio	P-value
**Patients**	31	155		
**Hospitalized**	<20	<20	0.64	.57
**Ventilated**	0	<20	n/a	>.99
**Deceased**	0	<20	n/a	>.99

Table S7. Immunohistochemistry reagents.

Antigen	Antibody Clone	Dilution	Source
**Ym1**	Goat polyclonal – Biotinylated	1:100	R&D – BAF2446
**Relmα**	Rabbit polyclonal	1:100	Peprotech – 500-P214
**Hyaluronan binding protein**	Biotinylated	1:100	Merck Millipore - 385911
-	Streptavidin 557	1:800	R&D – NL999
-	Donkey anti-rabbit IgG 637	1:200	R&D – NL005
